# Neither *per*, nor *tim1*, nor *cry2* alone are essential components of the molecular circadian clockwork in the Madeira cockroach

**DOI:** 10.1371/journal.pone.0235930

**Published:** 2020-08-04

**Authors:** Achim Werckenthin, Jannik Huber, Thordis Arnold, Susanne Koziarek, Marcus J. A. Plath, Jenny A. Plath, Olaf Stursberg, Hanspeter Herzel, Monika Stengl

**Affiliations:** 1 Department of Animal Physiology/Neuroethology, University of Kassel, Kassel, Germany; 2 Department of Control and System Theory, University of Kassel, Kassel, Germany; 3 Department of Theoretical Biology, Charité Berlin, Berlin, Germany; Biocenter, Universität Würzburg, GERMANY

## Abstract

Circadian clocks control rhythms in physiology and behavior entrained to 24 h light–dark cycles. Despite of conserved general schemes, molecular circadian clockworks differ between insect species. With RNA interference (RNA_i_) we examined an ancient circadian clockwork in a basic insect, the hemimetabolous Madeira cockroach *Rhyparobia maderae*. With injections of double-stranded RNA (dsRNA) of cockroach *period* (*Rm´per*), *timeless 1* (*Rm´tim1*), or *cryptochrome 2* (*Rm´cry2*) we searched for essential components of the clock´s core negative feedback loop. Single injections of dsRNA of each clock gene into adult cockroaches successfully and permanently knocked down respective mRNA levels within ~two weeks deleting daytime-dependent mRNA rhythms for *Rm´per* and *Rm´cry2*. *Rm´per*^*RNAi*^ or *Rm´cry2*^*RNAi*^ affected total mRNA levels of both genes, while *Rm´tim1* transcription was independent of both, also keeping rhythmic expression. Unexpectedly, circadian locomotor activity of most cockroaches remained rhythmic for each clock gene knockdown employed. It expressed weakened rhythms and unchanged periods for *Rm´per*^*RNAi*^ and shorter periods for *Rm´tim1*^*RNAi*^ and *Rm´cry2*^*RNAi*^.As a hypothesis of the cockroach´s molecular clockwork, a basic network of switched differential equations was developed to model the oscillatory behavior of clock cells expressing respective clock genes. Data were consistent with two synchronized main groups of coupled oscillator cells, a leading (morning) oscillator, or a lagging (evening) oscillator that couple via mutual inhibition. The morning oscillators express shorter, the evening oscillators longer endogenous periods based on core feedback loops with either PER, TIM1, or CRY2/PER complexes as dominant negative feedback of the clockwork. We hypothesize that dominant morning oscillator cells with shorter periods express PER, but not CRY2, or TIM1 as suppressor of clock gene expression, while two groups of evening oscillator cells with longer periods either comprise TIM1 or CRY2/PER suppressing complexes. Modelling suggests that there is an additional negative feedback next to Rm´PER in cockroach morning oscillator cells.

## Introduction

The molecular circadian clockwork [1] that controls rest-activity rhythms in insects is studied best in the fruitfly *Drosophila melanogaster* [2]. It is built of several interlocked transcriptional/posttranscriptional feedback loops resulting in circadian oscillations of mRNA- and protein levels. Part of the core feedback loop are the transcription factors CLOCK (Dm’CLK) and CYCLE (Dm’CYC). During the middle of the day to the early night, they activate the transcription of the E-box containing clock genes *period* (*Dm’per*) and *timeless* (*Dm’tim*). Both gene products, Dm’PER and Dm’TIM, inhibit their own transcription during the late night reducing respective mRNA levels until the early day. Thereby, mRNA levels of *Dm’per* and *Dm’tim* rise with endogenous circadian rhythmicity during the late day until early evening, before they decline again during the late night. About 6–8 h after their mRNA peaks the clock proteins Dm’PER and Dm’TIM accumulate during the middle of the night. The delay or phase difference between the rhythms in mRNA- and clock protein accumulation are regulated via consecutive, interlinked phosphorylations [2]. Phosphorylation-dependently, Dm’PER/Dm’TIM heterodimers translocate to the nucleus and inhibit their own transcription via interaction with Dm’CLK/Dm’CYC heterodimers. The rate of accumulation is regulated further light-dependently via CRYPTOCHROME 1 (Dm’CRY1). Dm’CRY1 in the fruitfly functions as blue light-sensitive photopigment that initiates light-dependent degradation of Dm’TIM. When Dm’PER is not protected via heteromerization with Dm’TIM, either in the cytoplasm or in the nucleus, Dm’PER is degraded also [2, 3]. When protected via heteromerization, however, they can accumulate in the cytoplasm and move to the nucleus to act as transcriptional inhibitors until Dm’PER protein is degraded phosphorylation- and Dm’CRY1/TIM-dependently. Thus, a new cycle of transcription starts during the day [2].

Whereas the principle elements recruited to form the molecular circadian clockwork are identical in different insect species, there are striking differences upon closer observation [4]. There are two types of cryptochromes [3]. While the *Drosophila*-type CRY1 acts as non-visual photopigment, the mammalian-type CRY2 acts as transcriptional repressor in the circadian core feedback loop. Furthermore, there are two types of *tim* genes with TIM1 being the transcriptional repressor, possibly originating from a duplication of *timeout* (*tim2*) [5, 6]. In hymenopterans such as the honey bee *Apis mellifera Am’tim1* is missing from the genome and Am’CRY2 functions as transcriptional repressor [5]. Depending on the insect species, either *cry1* or *cry2*, or both are present, as well as *tim1* or *tim2*, or both can be found in the genome [4]. Since *per*, *tim1*, and *cry2* are present together in most basic insects such as the cricket *Gryllus bimaculatus* and the Madeira cockroach [7–9, 4], this appears to be the ancestral form of the core-feedback loop from which other core loops were derived as in *D*. *melanogaster* and *A*. *mellifera*. Furthermore, the importance of *tim1* and *cry2* as negative regulators of the feedback loop for maintaining circadian rhythmicity also differs between insects [4]. *D*. *melanogaster Dm’tim1* knockout mutants are arrhythmic [10], as are individuals of the primitive insect *Thermobia domestica*, in which *Td’tim1* transcript was down-regulated [11]. In contrast, *tim1* appears to be expendable for circadian rhythmicity in the cricket *G*. *bimaculatus*, although *Gb’tim1* knockdown shortens circadian rhythms in this species [7].

The Madeira cockroach *Rhyparobia maderae (synonym*: *Leucophaea maderae*) is an established model organism in chronobiology, especially suited to behavioral, cellular, and electrophysiological analysis [12, 13]. While its genome is not available, exploiting transcriptomics and RNA interference (RNA_i_) mechanism by injecting double-stranded RNA (dsRNA) is a simple method to analyze the function of genes. The RNA_i_ method appears to be especially potent in hemimetabolous insects [14]. Here, we used RNA_i_ to examine the function of three circadian genes of the core-feedback loop system in *R*. *maderae*: *Rm´per*,*Rm´tim1*, and *Rm´cry2* that all were suggested to be negative feedback regulators. Furthermore, to challenge our interpretations of the role of single components of the circadian clockwork in the Madeira cockroach quantitative modelling was employed.

## Material and methods

### Cloning and dsRNA synthesis

The plasmid used for the *Rm´per* and *Rm´tim1* dsRNA templates was described in a previous paper [8]. The complete open reading frame of *Rm´cry2* was amplified from a brain cDNA library using a polymerase enzyme mix (High Fidelity PCR Enzyme Mix, Thermo Scientific, Waltham, MA; primers (S1 Table) with the following program: 2 min 94°C; 5 cycles: 30 s 94°C, 30 s 50°C, 150 s 72°C; 15 cycles: 30 s 94°C, 30 s 50°C, 150 s 72°C; 15 cycles: 30 s 94°C, 30 s 45°C, 150 s 72°C; 300 s 72°C). Amplicons were then TA cloned into pGEMT-easy, using the kit supplied with the plasmid (Promega, Fitchburg, WI). Primers with a T7 overhang (S1 Table) were used to amplify DNA templates for in-vitro transcription. Amplicons spanned 800 bp (*Rm´per*, JX235363), 591 bp (*Rm´tim1*, JX266619*)*, 791 bp (*Rm´cry2*, JX266618*)*, and 504 bp (*gfp*, L29345) of the genes, respectively. The MEGAscript T7 Transcription Kit (Thermo Fisher Scientific, Waltham, MA) was used to transcribe single-stranded RNA strands, which were subsequently extracted using Roti-Phenol/Chloroform/Isoamylalkohol (Carl Roth, Karlsruhe, Germany), washed two times with chloroform and precipitated using isopropanol. The pellet was washed two times with 80% ethanol, dried and dissolved in TE buffer. Equimolar amounts of sense and antisense RNA were then combined, denatured at 99°C for 5 min and incubated at room temperature for 15 min. The dsRNA was then extracted as described before and dissolved in ddH_2_O.

### Animal rearing and behavioral experiments

All *R*. *maderae* used in this study were taken from inbred mass cultures of the University of Kassel. Illumination in these colonies was approximately 100 lx for animals at all stages with a 12 h photoperiod (LD12:12) from a cold-white strip light. They were fed three times a week with dog food (Happydog Flocken Mixer, Interquell, Großaitingen, Germany), apples and carrots, water was supplied *ad libitum*. Only adult male cockroaches were used for the experiments. Prior to the experiments they were kept for at least one week in constant darkness (DD) in the running wheels to monitor activity. They were fed with rodent chow (ssniff V2144, Soest, Germany) and water ad libitum. Only animals showing rhythmic activity for at least one week were used for subsequent experiments (n = 55). Animals were stunned with CO_2_, then, for RNA interference (RNA_i_) experiments 12 μg of double-stranded RNA (dsRNA) in 10 μl ddH_2_O were injected into the hemolymph below the membrane between coxa and thorax using a glass capillary (S1 Fig). As controls dsRNA of green fluorescent protein (GFP) was injected (n = 13). GFP is not present in the Madeira cockroach genome. Animals were monitored for one month after dsRNA administration. Since animals were often inactive right after the injection and RNA_i_ took a while to take effect, the first two weeks after each injection were not used for statistical analysis. For evaluation of dsRNA-dependent activity changes, activity before and after injection was compared in the same animal during the week before injection, and in the interval of 3^rd^-4^th^ week after injection (for exceptions see S2 Table; *Rm’per* n = 12; *Rm’tim1* n = 10; *Rm’cry2* n = 20).

### Time series

For the time series experiments, 9 animals per time point were injected as described above for the behavioral experiments and kept in LD12:12 at 100 lx for one month after injection. They were sacrificed at the respective time points (Zeitgeber time = ZT) indicated. Per ZT the supraesophageal ganglia of three animals each were pooled, and each of the three pools per timepoint were measured in triplicates in the quantitative real-time polymerase chain reaction (qPCR) analysis.

### Quantitative PCR

The supraesophageal ganglion was removed and snap frozen in liquid nitrogen. Total RNA was extracted using the RNeasy Mini Kit (Qiagen, Vento, Netherlands) according to the manufacturer’s protocol. Putative DNA contamination was removed using ~1U/μg DNase I (Thermo Fisher Scientific, Waltham, MA) and DNase was inactivated adding 10 mM EDTA and incubating at 65°C for 10 min. The SensiFAST SYBR No-ROX One-Step Kit (Bioline, London, UK) was used according to the manufacturer’s protocol with a Mastercycler ep realplex (Eppendorf, Hamburg, Germany) to perform qPCR. *Rm’rpl18* (MT524704) was used as reference gene. Single amplicons were confirmed with a melting-curve analysis and data were analyzed using the standard 2^-ΔΔCT^ method [15] where all ΔCT values of one gene of interest were normalized against the mean ΔCT of the respective gene in the control group.

### Statistical analysis

**Fig 1:**
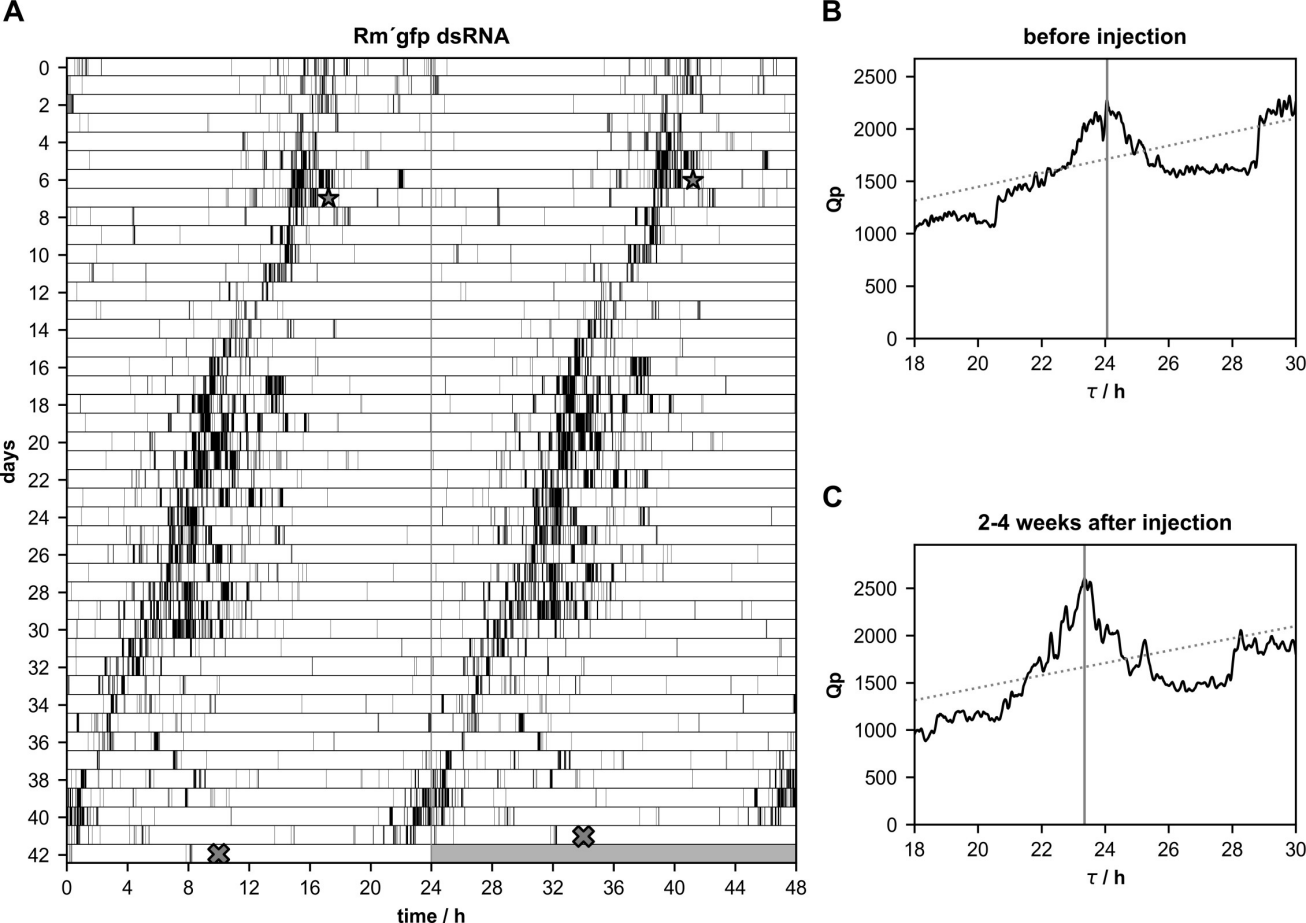
**A-C.** Control injections of *gfp* double stranded RNA (*gfp* dsRNA) did not change the circadian period(τ) of rhythmic running wheel activity in Madeira cockroaches (n = 10 of 13). **(A)** Double-plotted running wheel activity of a Madeira cockroach in constant darkness revealed only small, random changes in its period during the course of the 42 days long recording. An example plot (total n = 13) is shown. At day 7 (star) *gfp* dsRNA was injected, the animal was sacrificed at day 42 (cross). Chi-square periodogram analysis of the number of running wheel turns indicated significant rhythmicity with a period of 24.07 h 1–7 days before *gfp* dsRNA injection **(B)** and 23.35 h 2–4 weeks after the injection **(C)**.

**Fig 2:**
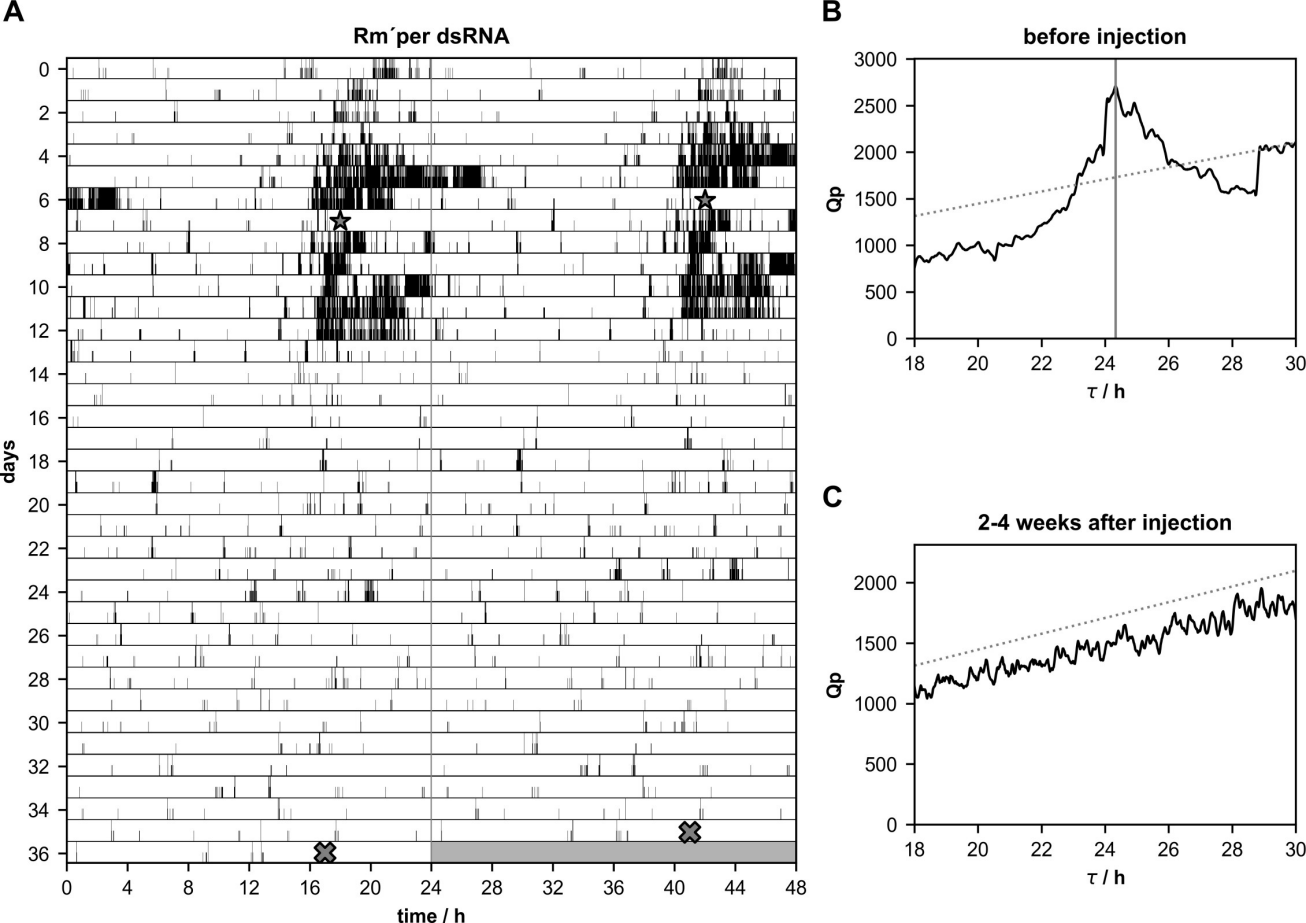
**A-C.** Injections of *Rm´per* dsRNA abolished circadian rhythmicity of running wheel locomotor activity in some Madeira cockroaches (n = 4 of 12). **(A)** Double-plotted running wheel activity of a Madeira cockroach in constant darkness 7 days before, and 29 days after the injection of *Rm´per* dsRNA (star). At day 36 of the locomotor activity recording the cockroach was sacrificed (cross) and qPCR was used to confirm the knockdown. Chi-square periodogram analysis of the number of running wheel turns indicated significant rhythmicity with a period(τ)of 24.33 h 1–7 days before *Rm´per* dsRNA injection **(B)** and loss of rhythmicity 2–4 weeks after the injection **(C)**.

**Fig 3:**
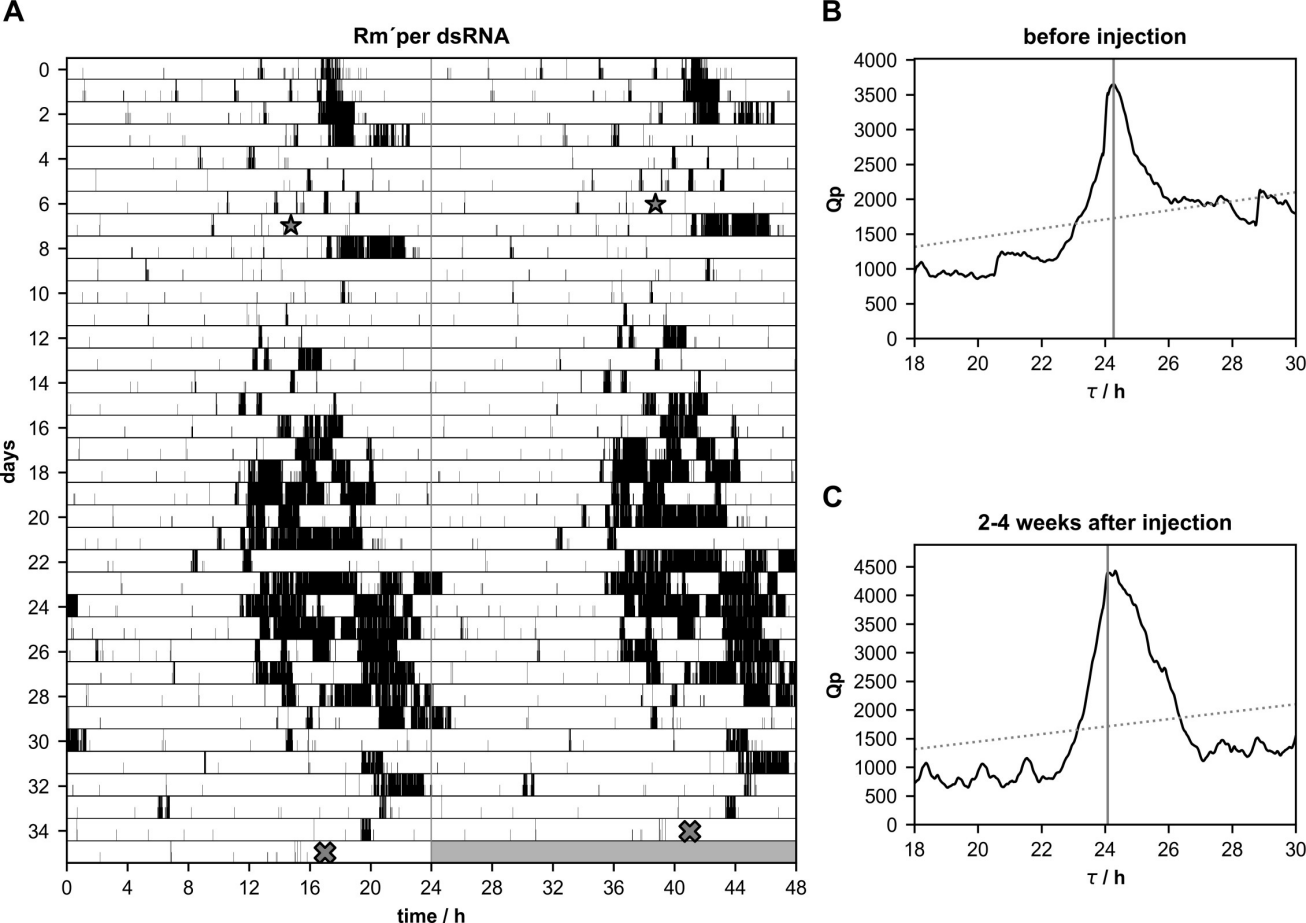
**A-C.** After injections of *Rm´per* dsRNA circadian rhythmicity of running wheel activity remained synchronized with almost unchanged period (τ)in only one Madeira cockroach (n = 1 of 12). **(A)** Double-plotted running wheel activity rhythm of a Madeira cockroach in constant darkness one week before, and 28 days after the injection of *Rm´per* dsRNA (star). At day 35 of the locomotor activity recording the cockroach was sacrificed (cross) and qPCR was used to confirm the knockdown. Chi-square periodogram analysis of the number of running wheel turns indicated significant rhythmicity with a period of 24.27 h 1–7 days before *Rm´per* dsRNA injection **(B)** and 24.08 h 2–4 weeks after the injection **(C)**.

**Fig 4:**
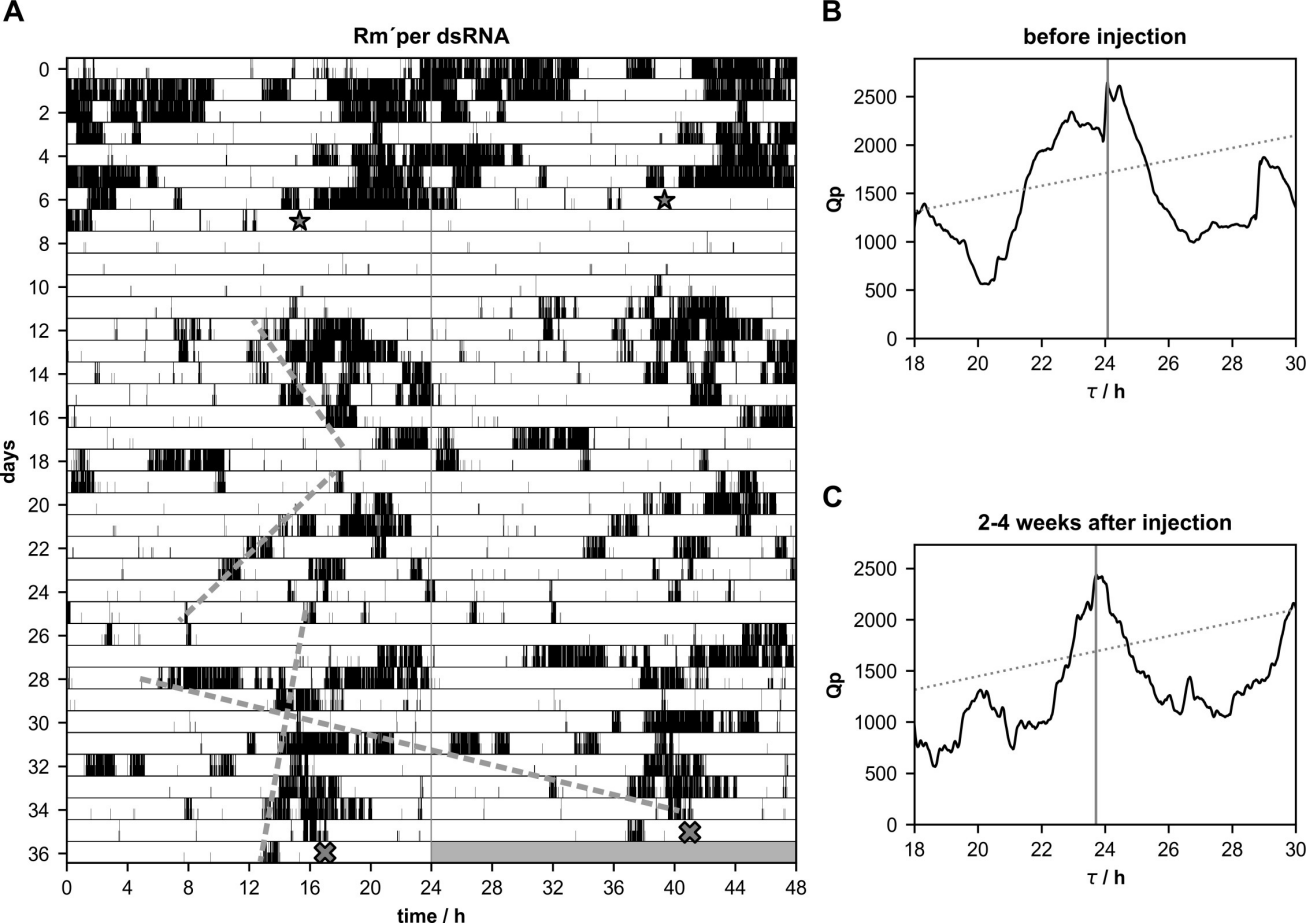
**A-C.** Most Madeira cockroaches retained circadian rhythmicity (n = 8 of 12), but expressed weaker (n = 6 of 12) and/or desynchronized rhythms (n = 3 of 12) with more than one period(τ) after injections of *Rm´per* dsRNA. **(A)** Double-plotted running wheel activity of a Madeira cockroach in constant darkness 7 days before, and 29 days after the injection of *Rm´per* dsRNA (star). At day 36 of the locomotor activity recording the cockroach was sacrificed (cross) and qPCR was used to confirm the knockdown. Chi-square periodogram analysis of the number of running wheel turns indicated significant rhythmicity with a period of 24.08 h 1–7 days before *Rm´per* dsRNA injection **(B)** and 23.72 h 2–4 weeks after the injection **(C)**. Already before injection the broad, two peaked distribution of significant rhythmicity indicated only loosely coupled oscillators controlling locomotor rhythms in this individual cockroach. Knockdown of *Rm´per* mRNA further dissociated underlying short and long rhythmic components (dashed lines), but did not completely delete rhythmicity over the course of 4 weeks.

**Fig 5:**
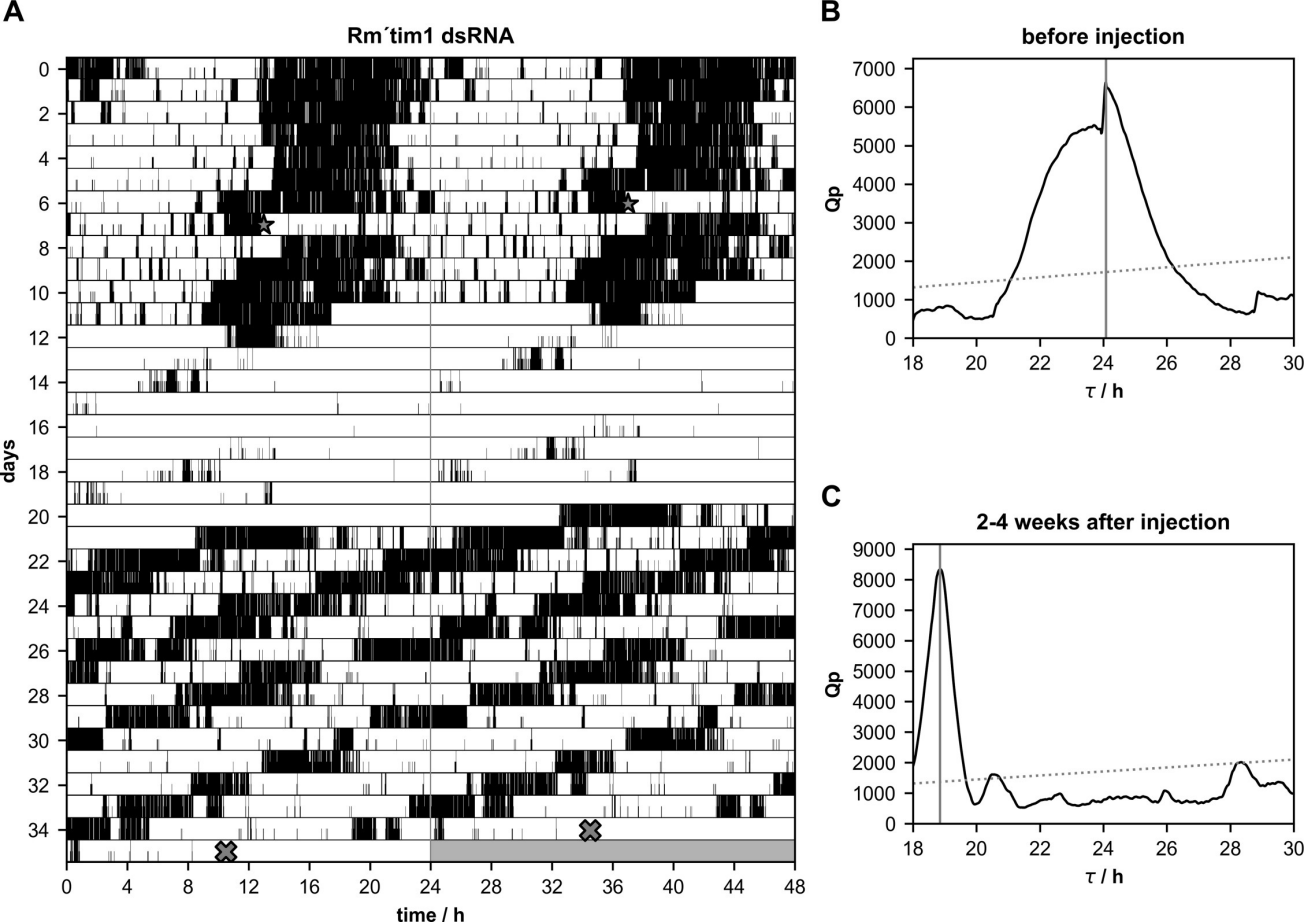
**A-C.** Injections of *Rm´tim1* dsRNA significantly shortened the free-running period(τ) of circadian locomotor activity rhythms (n = 8 of 10; p = 0.0016). **(A)** Double-plotted running wheel activity of a Madeira cockroach in constant darkness. *Rm´tim1* dsRNA was injected at day 7 (star) of the recording. The injected cockroach retained synchronized circadian locomotor activity rhythms, but with a significantly shortened period. At day 35 of the locomotor activity recording the cockroach was sacrificed (cross) and qPCR was used to confirm the knockdown. Chi-square periodogram analysis of the activity shown in **(A)**, 1–7 days before dsRNA injection revealed a period of 24.08 h **(B)** and a period of 18.85 h 2–4 weeks after the injection **(C)**.

**Fig 6:**
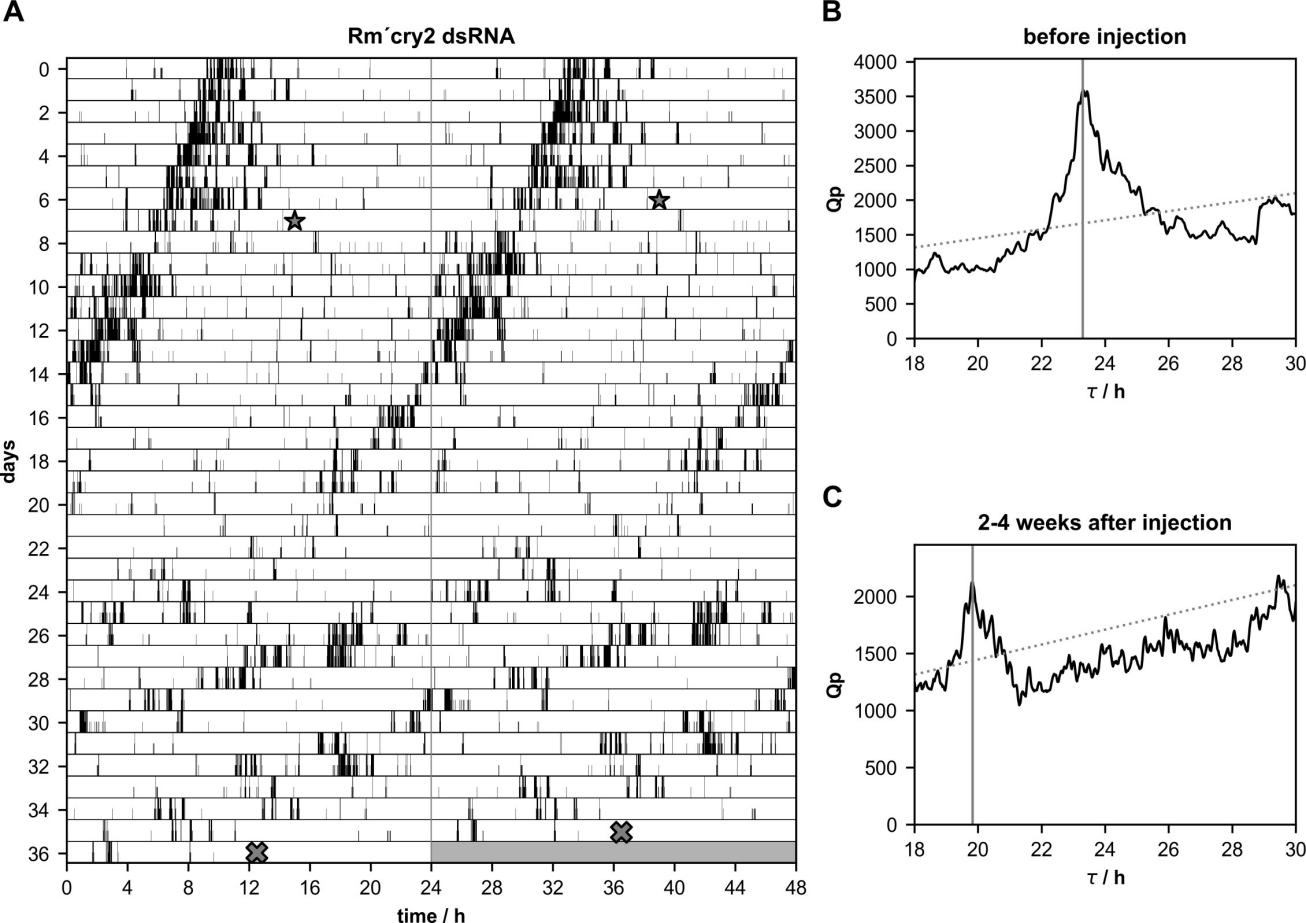
**A-C.** Injections of *Rm´cry2* dsRNA significantly shortened the free-running period(τ)of locomotor activity rhythms (n = 14 of 20; p<0.0001). **(A)** Double-plotted running wheel activity of a Madeira cockroach in constant darkness revealed significant shortening in the period of its locomotor activity rhythm after injection of *Rm´cry2* dsRNA at day 7 (star) of the recording. At day 36 of the locomotor activity recording the cockroach was sacrificed (cross) and qPCR was used to confirm the knockdown. Chi-square periodogram analysis of the activity 1–7 days before dsRNA injection revealed a period of 23.30 h **(B)**, and 2–4 weeks after the injection of 19.83 h **(C)**.

Analysis and data visualization of the behavioral and time series experiments were performed with Python 3.7.4 using the numpy [16], scipy [17] and matplotlib [18] packages and R 3.5.1 using R Markdown with R Studio 1.2.5033 and the tidyverse [19], reticulate [20], xsp [21], nlme [22], multcomp [23], and lsmeans [24] packages. The statistical significance level was set to 0.05, if not stated otherwise. To evaluate rhythmicity in behavioral experiments, chi-square periodogram analysis of the activity was performed during the first week before the dsRNA injection and in the 3^rd^-4^th^ week after the injection. Periodograms were smoothed with second-order Savitzky-Golay filter (window size 11) and applied to periods (τ) from 18 to 30 h. Any peak over the significance level (dotted line in Figs 1–6; p <0.000001) was counted as rhythmic activity with respective period. Arrhythmic activity was defined as the absence of significant peaks in the chi-square periodogram analysis. Desynchronization was determined when more than one peak occurred over the significance level with a period difference of at least 1 h and when at the same time more than one rhythmic component was detectable (by visual inspection, as in Fig 4, dashed lines) in the locomotor activity blots after the injection. For this, in addition to the analysis of 3^rd^-4^th^ week, segments of several days were selected manually and analyzed with the chi-square periodogram (S2 Table). The circadian period τ was defined as the most prominent peak over the significance level. For comparison of τ before with τ after the injection, as well as to compare Δτ (τ_after_−τ_before_) between the control and each experimental group, two-tailed Student’s t-tests were performed. In behavioral experiments, differences in relative mRNA expression ratios of the respective gene of interest between control and experimental group were evaluated with a Kruskal-Wallis test.

For time series experiments, ZT-dependent differences in mRNA expression levels of individual genes of interests were analyzed for wildtype, *Rm’tim1*, and *Rm’cry2* knockdowns. As pointed out in Boisgontier and Cheval [25], linear mixed models (LMM)–opposed to ANOVA or repeated measure ANOVA–can account for sampling variability of random samples independently of investigated effects (fixed effects). Accordingly, since the selected animals for this experiment only represented a random sample from the whole possible population of animals, we employed LMMto account for this variation in our data. The computational methods and model formulation used (nlme package for R; [22]) are based on the works of Lindstrom and Bates [26] and Laird and Ware [27]. For each time series, we compared the mRNA expression level of each ZT with the lowest mRNA expression level found in that time series (fixed effect in the model). We compensated for sample variation by using the sample ID as random variable in the LMM. Furthermore, to evaluate knockdown-dependent effects on mRNA expression levels in the time series, data for all ZTs of a gene of interest were pooled and compared with the equally treated corresponding wildtype data using the same LMM approach. The size of the data set allowed no ZT-dependent multiple comparison between knockdown and wildtype group.

## Results

In the Madeira cockroach only the circadian clock genes *period* (*Rm´per*), *timeless1* (*Rm´tim1*), and *cryptochrome2* (*Rm´cry2*) were described so far that all expressed circadian rhythms in their expression level [8]. We investigated whether the circadian clock proteins PER, TIM1, and CRY2 in *R*. *maderae* (Rm´PER, Rm´TIM1, Rm´CRY2)play essential roles as negative feedback regulators in the circadian core clockwork that control circadian locomotor activity rhythms of the Madeira cockroach. RNA interference (RNA_i_) experiments were performed using systemically injected double stranded RNA (dsRNA) to knock down respective mRNA and protein levels. Since an intact molecular circadian clockwork is assumed as prerequisite to circadian locomotor activity rhythms, we combined RNA_i_ experiments with running wheel assays. In the fruitfly *Drosophila melanogaster* molecular circadian clockwork, both, PER and TIM1, but not CRY2 are essential clockwork components for circadian locomotor activity rhythms. In the molecular clockwork of mammals, PERs and also CRY2, but not TIM1 are essential negative feedback regulator proteins of the core clock feedback loop. Thus, we expected that knockdown of the mRNA of *Rm´per* and either *Rm´cry2* or *Rm´tim1* genes would impair the core feedback loop of the molecular circadian clock, leading to arrhythmic behavior. Alternatively, comparably to crickets we expected that knockdown of the mRNA of *Rm´per*, and *Rm´cry2*, but not of *Rm´tim1* alone hits essential negative feedback loops of the core clock deleting circadian locomotor activity rhythms [28, 7, 29].

### Neither *Rm´per*, *Rm´tim1*, nor *Rm´cry2* dsRNA injected cockroaches became arrhythmic

Male cockroaches were kept in running wheels in constant darkness (DD) to monitor their circadian locomotor activity rhythms, to select a population of rhythmic cockroaches. After about one week of rhythmic activity dsRNA injections were performed to knock down mRNA levels of *Rm´per* (n = 12),*Rm´tim1* (n = 10), or *Rm´cry2* (n = 20). Furthermore, injections of dsRNA of *gfp*, a gene that does not occur in insects, were employed as controls (n = 13). Of 13 *gfp* dsRNA injected cockroaches 10 kept their rhythmicity, while 3 strongly reduced their activity, appearing arrhythmic after the injection. While spontaneous changes in period could occur in the locomotor activity rhythms in these control cockroaches, the changes were small and did not correlate with the injections of dsRNA of *gfp* (Fig 1; Table 1; p = 0.7108). After injections of *Rm´per* dsRNA a few cockroaches (n = 4 of 12) became arrhythmic (Fig 2; Table 2), while 8 cockroaches retained rhythmic behavior. One of the 8 rhythmic cockroaches expressed strong, synchronized circadian locomotor activity rhythm with almost unchanged period, despite of successful dsRNA-dependent knockdown of *Rm´per* expression (Fig 3; Table 2). Synchronized circadian activity rhythms meant that only one rhythmic component (only one peak over the significance threshold in chi square periodogram analysis) was apparent at the same time in the behavioral rhythms despite its control via different neuronal circuits in both optic lobes, as well as within one optic lobe. Of the remaining 7 rhythmic cockroaches 3 expressed at least in part desynchronized rhythms with more than one peak over the significance threshold in chi square periodogram analysis (Fig 4; Table 2; please see Material and Methods). Of these 7 rhythmic cockroaches 1 expressed strong (Fig 4) and 6 expressed only weak rhythms. In a comparison of periods before and after the injection the *Rm´per* dsRNA injected cockroaches showed no significant period changes as compared to the controls (Table 1; n = 8 of 12; p = 0.4579).

**Table 1:** Periods (τ)of free-running locomotor activity rhythms.

τ / h ± SD; number			
dsRNA	1–7 days before injection	3–4 weeks after injection	Difference / after–before injection^#^
*gfp (control)*	23.42 ± 1.64; 13	23.61 ± 1.82; 10	0.22 ± 1.80
*Rm’per*	23.59 ± 0.81; 12	24.32 ± 0.93; 8	0.76 ± 0.93
*Rm’tim1*	23.36 ± 1.92; 10	19.42 ± 0.63; 8^‡^	-3.82 ± 2.17^‡^
*Rm’cry2*	23.79 ± 0.44; 20	20.98 ± 1.61; 14^‡^	-2.83 ± 1.69^‡^

^#^ only animals with determinable period after injection were included.

^†^ period differed highly significantly between evaluated time intervals before and after injection; *Rm’tim1*: p = 0.0016; *Rm’cry2*: p<0.0001, paired Student’s t-test.

^‡^ period differed highly significantly from *gfp* control dsRNA injected animals; *Rm’tim1*: p = 0.0005, *Rm’cry2*: p = 0.0003, Student’s t-test.

**Table 2:** Rhythmicity of free-running locomotor activity rhythms in controls and experimental animals.

dsRNA	Strongly rhythmic / n		Weakly rhythmic / n		Arrhythmic / n	Total / n
	Syn-chronized	Desyn-chronized	Syn-chronized	Desyn-chronized		
***gfp (control)***	2	2	5	1	3	13
***Rm’per***	1	1	4	2	4	12
***Rm’tim1***	1	3	2	2	2	10
***Rm’cry2***	3	1	6	4	6	20

In contrast to *Rm´per* dsRNA injections the injections of *Rm´tim1* dsRNA significantly shortened the period of the locomotor activity rhythm (Table 1; n = 8 of 10; p = 0.0005). While only 2 animals became arrhythmic after the injection and only 1cockroach maintained synchronized, strong rhythmicity with a stable shortened period 5 of the remaining 7 cockroaches also shortened their period but expressed more than one rhythmic component at least over a stretch of several days (Fig 5; Table 2). The changes in the period of the free-running locomotor activity rhythms started already in the first week after the injection. They remained throughout the course of most experiments that were stopped about 1 month after the injections.

**Fig 7:**
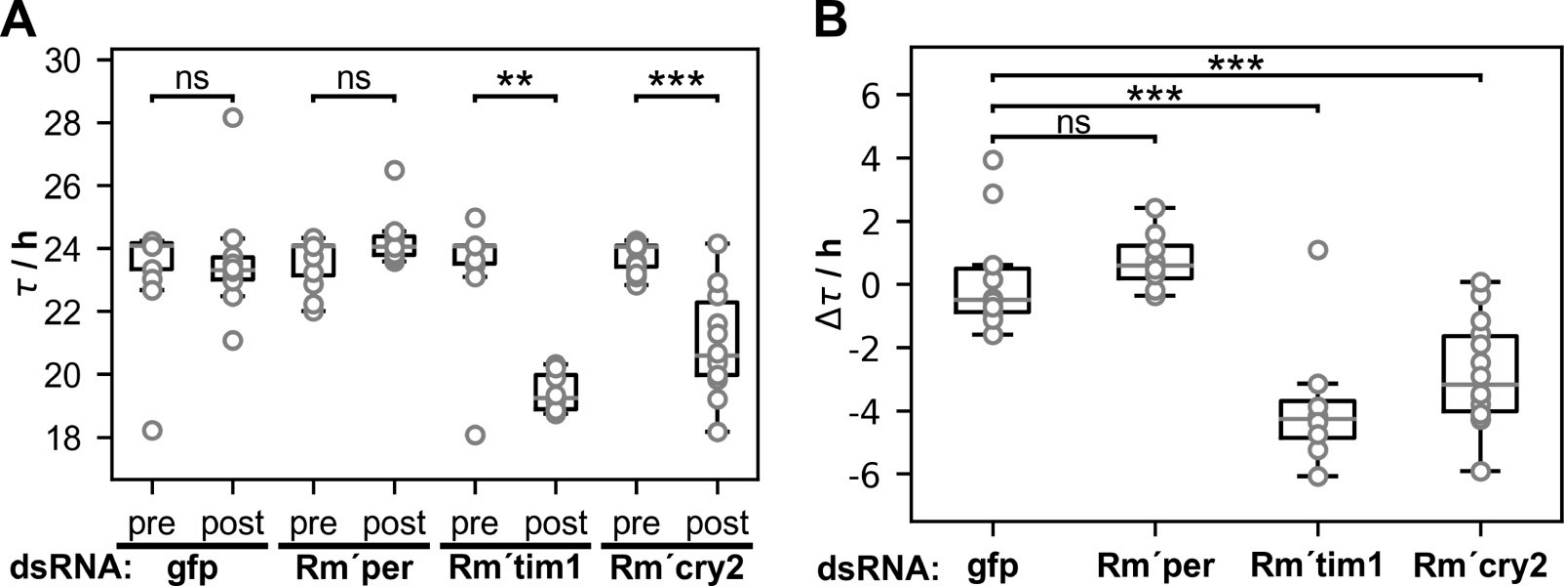
In running wheel assays *Rm´tim1* and *Rm´cry2* dsRNA injections, but not injections of *Rm´per* dsRNA significantly shortened the period (τ) of locomotor activity. (**A**)Comparison of period before and after injections of *gfp-* (n = 10; p = 0.7108), *Rm´per* (n = 8; p = 0.0546), *Rm´tim1*- (n = 8; p = 0.0016), or *Rm´cry2* dsRNA (n = 14; p<0.0001). Periods of dsRNA injected animals were measured 2–4 weeks after injection and compared to one week before the injection. After injections of *Rm´per* dsRNA cockroaches showed unchanged locomotor activity rhythms with only a tendency to develop longer periods, while both other injections shortened activity rhythms. (**B**)Comparison of the difference in periods of locomotor activity rhythms before and after injections (Δτ) between *gfp* controls (n = 10) and *Rm´per* (n = 8; p = 0.4579), *Rm´tim1*- (n = 8; p = 0.0005), or *Rm´cry2* dsRNA injected cockroaches (n = 14; p = 0.0003). Periods were determined using chi-square periodogram analysis. Student‘s t-tests were used to determine significant differences between animals of one group before and after the injection (**A**) and between Δτ of *gfp* control, the *Rm’per*, the *Rm´tim1*, and *Rm´cry2* dsRNA injected animals (**B**). ns: not significant; **: p<0.01; ***: p<0.001.

After injections of *Rm´cry2* dsRNA few cockroaches became arrhythmic (Table 2; n = 6 of 20). The majority (n = 14 of 20) remained rhythmic, but expressed shorter periods (Fig 6; Table 1; n = 14 of 20; p = 0.0003). Of the 14 rhythmic cockroaches4 expressed strong rhythmicity with a significantly shortened period of the locomotor activity rhythm throughout the recording time. The remaining 10 cockroaches showed weaker rhythms. Also, 5 of the 14 rhythmic cockroaches showed more than one rhythmic component over some days (Table 2). A comparison of periods before and after the injection of *Rm´tim1* or *Rm´cry2* dsRNA in all rhythmic cockroaches showed a significant shortening of the periods, also as compared to the controls (Fig 7; Table 1). In contrast, injection of *Rm´per* dsRNA did not significantly change the period of locomotor activity rhythms (Fig 7; Table 1). In summary, unexpectedly, despite of successful knockdown resulting in strong decreases in the gene products that became apparent already after one week, at least two thirds of the *Rm´per*, the *Rm´tim1*, and the *Rm´cry2* dsRNA injected cockroaches remained rhythmic in DD. However, there were differences between the three experimental groups. Injections of *Rm´per* dsRNA were weakening circadian locomotor activity rhythms without generating a significant change in the period (Fig 7; Tables 1 and 2). In contrast, both *Rm´tim1* and *Rm´cry2* dsRNA injections significantly shortened the rhythms´ period (Table 1).Thus, neither *Rm´per*, *Rm´tim1*, nor *Rm´cry2* alone appear to be essential for the circadian molecular clockwork of the Madeira cockroach in clock cells that control locomotor activity rhythms. Instead, locomotor rhythms appear to be controlled redundantly via clock neurons with either a short or a long period that are coupled.

### While RNA_i_-dependent knockdown of *Rm´per* and *Rm´cry2* affected each other, *Rm´tim1* knockdown was independent of both

**Fig 8:**
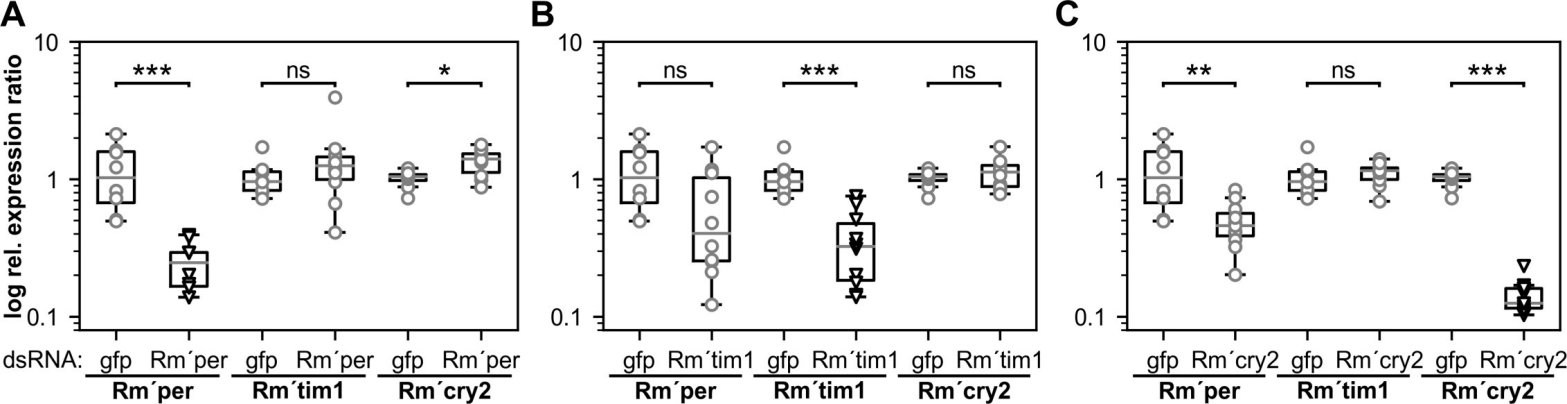
**A-C.** Quantitative PCR (qPCR) showed that *Rm´per* (A; n = 10), *Rm´tim1* (B; n = 10), and *Rm´cry2* (C; n = 11) dsRNA injection successfully downregulated mRNA levels of the respective gene (open triangles). The dsRNA experiments revealed only a significant interdependence of *Rm´cry2* with *Rm´per* transcript levels, while *Rm´tim1* was independent of both (**A-C**). Animals that were recorded before in the running wheel assays (Figs 1–6) were sacrificed about one month after injection of dsRNA and their mRNA levels were monitored using qPCR. Open circles and triangles indicate relative expression ratios of individual animals with respect to the mean value of *gfp* dsRNA injected animals (controls). Kruskal-Wallis tests were used to determine significant differences between *gfp* (n = 8) and *Rm’per/Rm´tim1*/*Rm´cry2* dsRNA injected animals. ns: not significant; *: p<0.05; **: p<0.01; ***: p<0.001.

Using qPCR about one month after dsRNA injections the success of the respective knockdown was examined for cockroaches tested before in the behavioral assays (Figs 1–6). Successful knockdown after only one injection of dsRNA in adult cockroaches appeared to persist for at least 6 months, apparently as long as animals survived. Thus, RNAi experiments are very successful and long-lasting for the examination of the role of specific gene products in the Madeira cockroach. Next to searching for a decrease in mRNA levels of the respectively dsRNA-targeted circadian clock gene it was also examined whether successful knockdown of either *Rm´per*,*Rm´tim1*, or *Rm´cry2* affected mRNA levels of any of the other not targeted clock genes when compared to *gfp* dsRNA (n = 8) injected control animals (Fig 8). Cockroaches were sacrificed at random circadian times (CTs) before examining mRNA levels of *Rm´per*, *Rm´tim1*, and *Rm´cry2* one month after the injection. Unexpectedly, we found that knockdown of *Rm´per* (n = 10; p = 0.0004) significantly increased *Rm´cry2* mRNA levels (Fig 8A; p = 0.0410), while knockdown of *Rm´cry2* (n = 11; p = 0.0003) significantly decreased *Rm´per* (Fig 8C; p = 0.0064), without affecting *Rm´tim1* mRNA levels (*Rm’per* dsRNA p = 0.2863; *Rm’cry2* dsRNA p = 0.2477). Accordingly, knockdown of *Rm´tim1* (n = 10; p = 0.0005) did not affect mRNA levels of either *Rm´per* (p = 0.0506) or *Rm´cry2* (Fig 8B; p = 0.4772). In summary, we concluded, that Rm´PER and Rm´CRY2 are expressed in the same clock cells, interacting with each other in the cell´s circadian core clockwork. In contrast, since Rm´TIM1 acts independently of both, it occurs in other clock cells. Furthermore, while Rm´PER appears to be a powerful inhibitor of transcription of both *Rm´per* and *Rm´cry2*, Rm´CRY2 rather decreases Rm´PER´s effectiveness as transcriptional inhibitor. Thus, we assume that there are at least three different groups of circadian oscillator neurons, either expressing Rm’PER alone, or Rm’TIM1 alone, or both Rm’PER and Rm’CRY2 together, as negative feedback loops of their respective molecular clockworks.

### The RNAi-dependent knockdown of *Rm´tim1* as well as of *Rm´cry2* mRNA abolished daytime-dependent cycling of mRNA levels of *Rm´per* and *Rm´cry2*, but not of *Rm´tim1*

**Fig 9:**
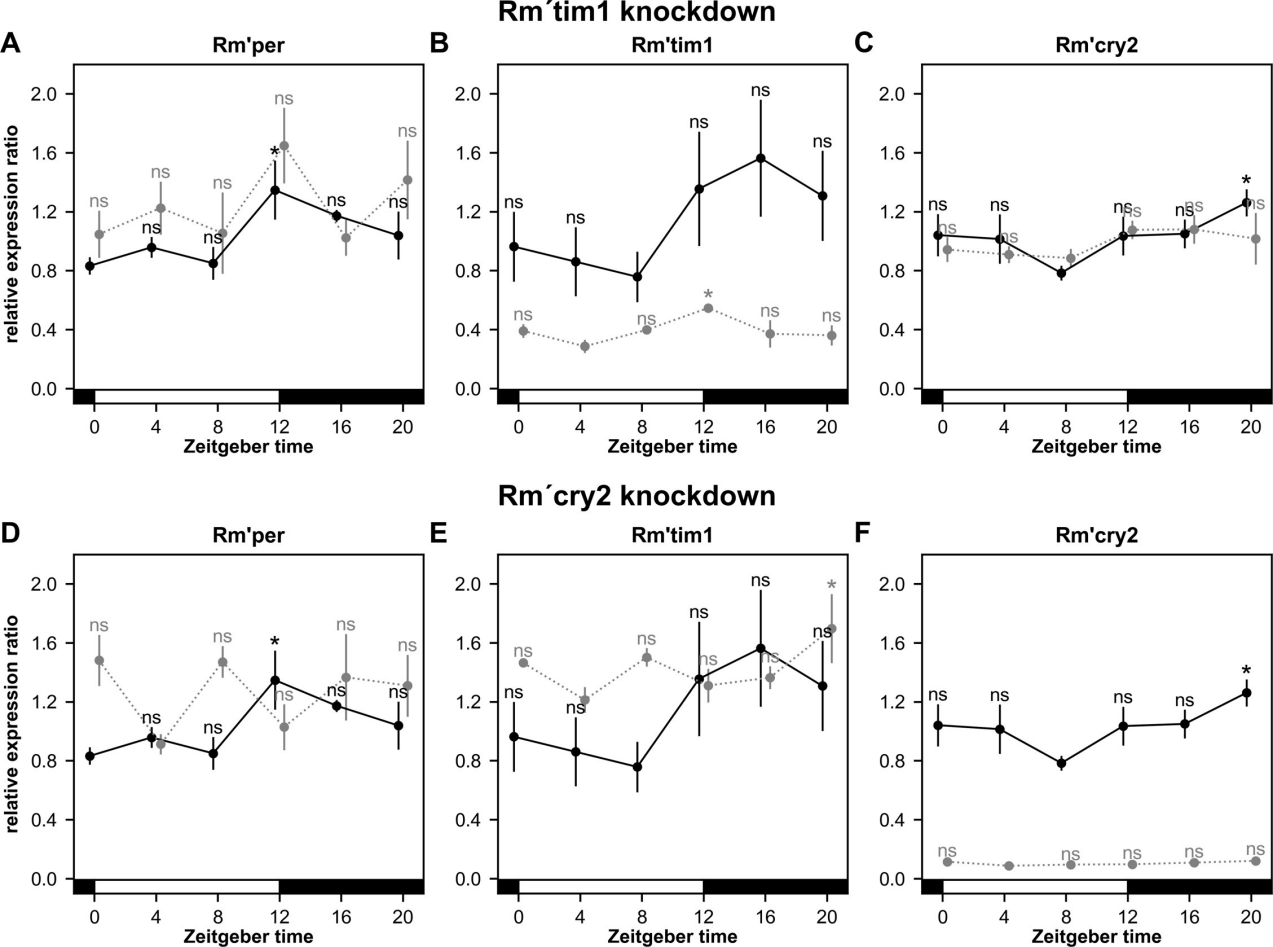
**A-F.** Except for *Rm´tim1* both, dsRNA-dependent downregulation of *Rm´tim1* (A-C) or of *Rm´cry2* (D-F) abolished cycling of mRNA levels of both other circadian clock genes examined. Solid lines represent control animals, dotted lines *Rm´tim1* (**A-C**) or *Rm´cry2* (**D-F**) dsRNA injected animals respectively. Relative expression ratios are analyzed compared to the lowest value of the respective curve. Relative expression ratios of *Rm´per* (**A**), and *Rm´cry2* (**C**),but not *Rm´tim1* (**B**)cycled ZT-dependently in controls (n = 3 per Zeitgeber time (ZT) in each group).Minima in mRNA levels of the controls are at ZT 0 (*Rm´per*) and at ZT 8 (*Rm´tim1*, *Rm´cry2*). Expression maxima in controls occurred at the beginning of the night (ZT 12; *Rm´per*), the middle of the night (ZT 16; *Rm´tim1*), or the end of the night (ZT 20; *Rm´cry2*).Successful knockdown of *Rm´tim1* to ~40% of WT mRNA levels deleted rhythmic expression of *Rm´per* (**A**) and *Rm´cry2* (**C**), but not of *Rm´tim1* (**B**). Knockdown of *Rm´cry2* to ~10% of WT levels deleted rhythmic expression of *Rm´per* (**D**) and *Rm´cry2* (**F**), but not of *Rm´tim1* (**E**). Whole brains of cockroaches at different ZTs in 12:12 LD cycles were collected for qPCR experiments. The bars at the bottom of the plots indicate light (white) and dark (black) phases. A linear mixed model was used to determine significant differences within groups. The ZT with the lowest data points within each curve was always compared with all other ZTs of the curve. Error bars represent standard errors. ns = not significant;*: p<0.05.

It was shown before that all three clock genes express circadian rhythms in their expression rate [8]. Neither knockdown of *Rm´tim1* nor *Rm´cry2* knockdown abolished behavioral rhythmicity, but strongly shortened τ of locomotor activity rhythms. Therefore, we expected that knockdowns did not delete daytime-dependent expression rhythms in all clock genes examined in LD. Thus, in another qPCR experiment it was examined whether daytime-dependent rhythms in the expression of the three circadian clock genes known in the Madeira cockroach were compromised via successful knockdown of either *Rm´tim1* (Fig 9A–9C; p<0.0001) or *Rm´cry2* (Fig 9D–9F; p<0.0001) mRNA levels (n = 3 pools per timepoint, 3 animals per pool, for each group).Knockdown of *Rm´tim1* reduced *Rm´tim1* mRNA levels to~40% of still rhythmically expressed mRNA levels (Fig 9B; p = 0.0191).Nevertheless, as compared to rhythmic controls (*Rm’per* p = 0.0313, *Rm’tim1* p = 0.1476,*Rm’cry2* p = 0.0402) dsRNA-dependent knockdown of *Rm´tim1* mRNA levels to ~40% deleted daytime-dependent rhythms of *Rm´per* and*Rm´cry2* mRNA levels (Fig 9A–9C;*Rm’per* p = 0.1236,*Rm’cry2* p = 0.2753). The *Rm´cry2* dsRNA injections knocked down *Rm´cry2* mRNA levels to ~10%, deleting rhythmic expression (Fig 9D; p = 0.0809). Also, dsRNA-dependent knockdown of *Rm´cry2* mRNA levels abolished daytime-dependent rhythms of *Rm´per* but not of *Rm´tim1* mRNA levels (Fig 9D–9F;*Rm’per* p = 0.0981, *Rm’tim1* p = 0.0374). In summary, RNA_i_-dependent knockdown of either *Rm´tim1* or *Rm´cry2* mRNA deleted rhythmic changes in the mRNA levels of all cockroach clock genes examined, except of *Rm´tim1*. Since we assumed that rhythmic expression of circadian clock genes is a prerequisite to rhythmic locomotor activity we concluded that loss in rhythmicity was due to desynchronization of otherwise rhythmic circadian clock neurons. We assumed that there are different, partly redundant ensembles of clock neurons expressing different molecular feedback loops that control rhythmic behavior in parallel. When only some of the molecular feedback loops were compromised via decreasing of respective mRNA levels, still there are clock cells left that expressed either short or long τ and that can drive rhythmic behavior. Interestingly, despite the remaining rhythmic expression of *Rm´tim1*, the ~60% decrease in mRNA levels compromised synchrony of other clock gene expressing clock cells, resulting in period shortening of locomotor activity rhythms. Thus, the concentration of clock gene products appears to be relevant for keeping synchrony.

### Modelling of core circadian feedback loops in the Madeira cockroach

**Fig 10:**
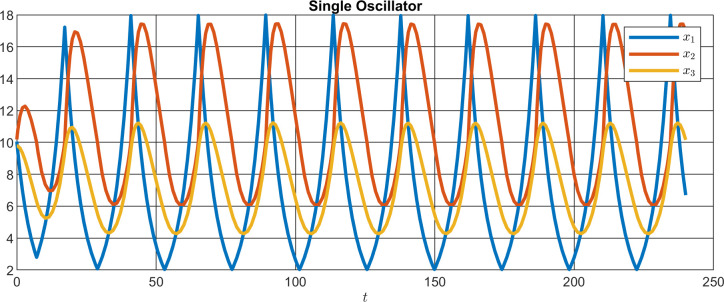
Simulation of single oscillator with three states modeled as Switching Linear System (SLS). SLS is a subclass of Hybrid Automata, combining continuous and discrete-valued dynamics.

**Fig 11:**
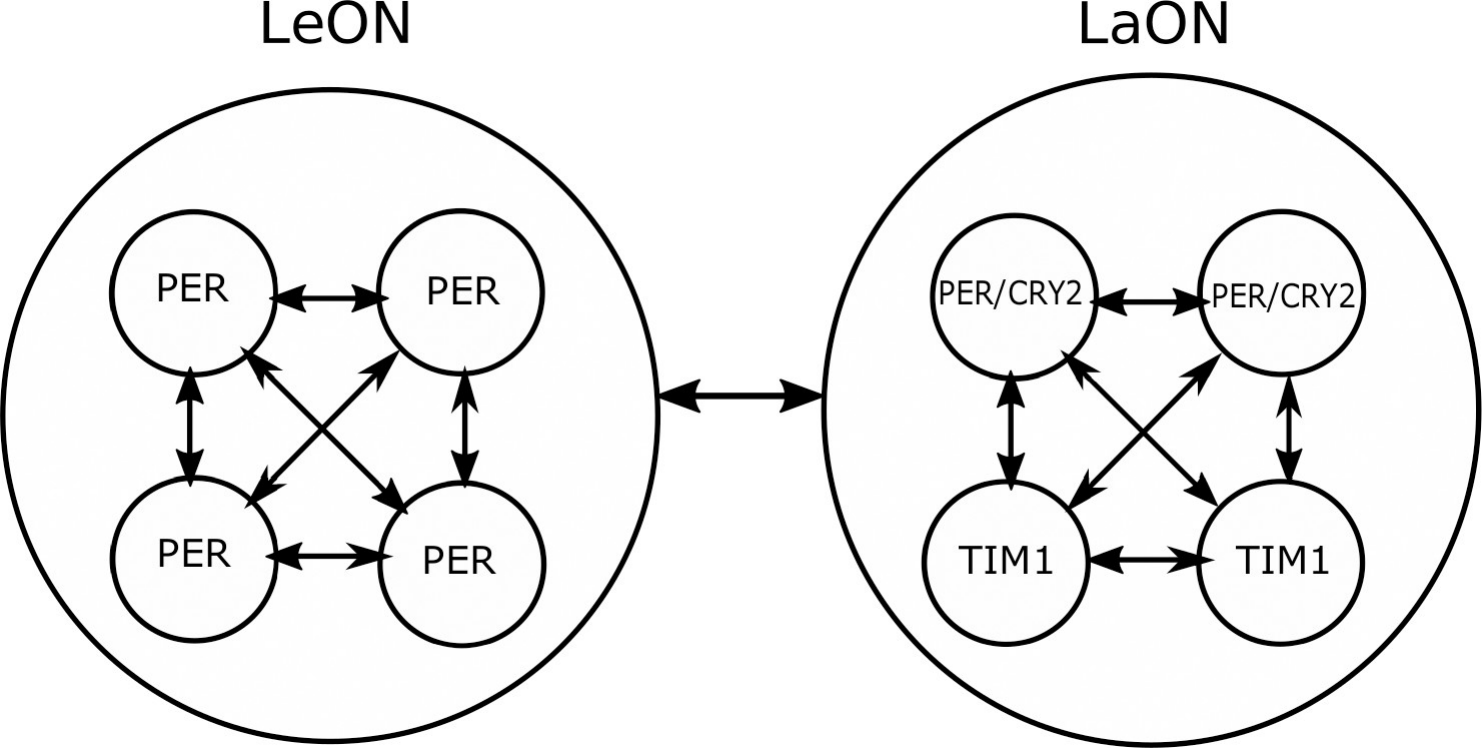
Two circadian oscillator networks with different molecular clockworks control locomotor activity rhythms of the Madeira cockroach. The lead oscillator network (LeON) with a shorter period and the lag oscillator network (LaON) with a longer period, each consist of four single oscillator neurons. In LeON only PERIOD (PER) constitutes the negative limb of the core transcriptional feedback loop in all of the circadian pacemaker neurons. However, in LaON two different cell types exist, one with PER and CRYPTOCHROME 2 (CRY2), and the other with TIMELESS1 (TIM1) as transcriptional repressors.

**Fig 12:**
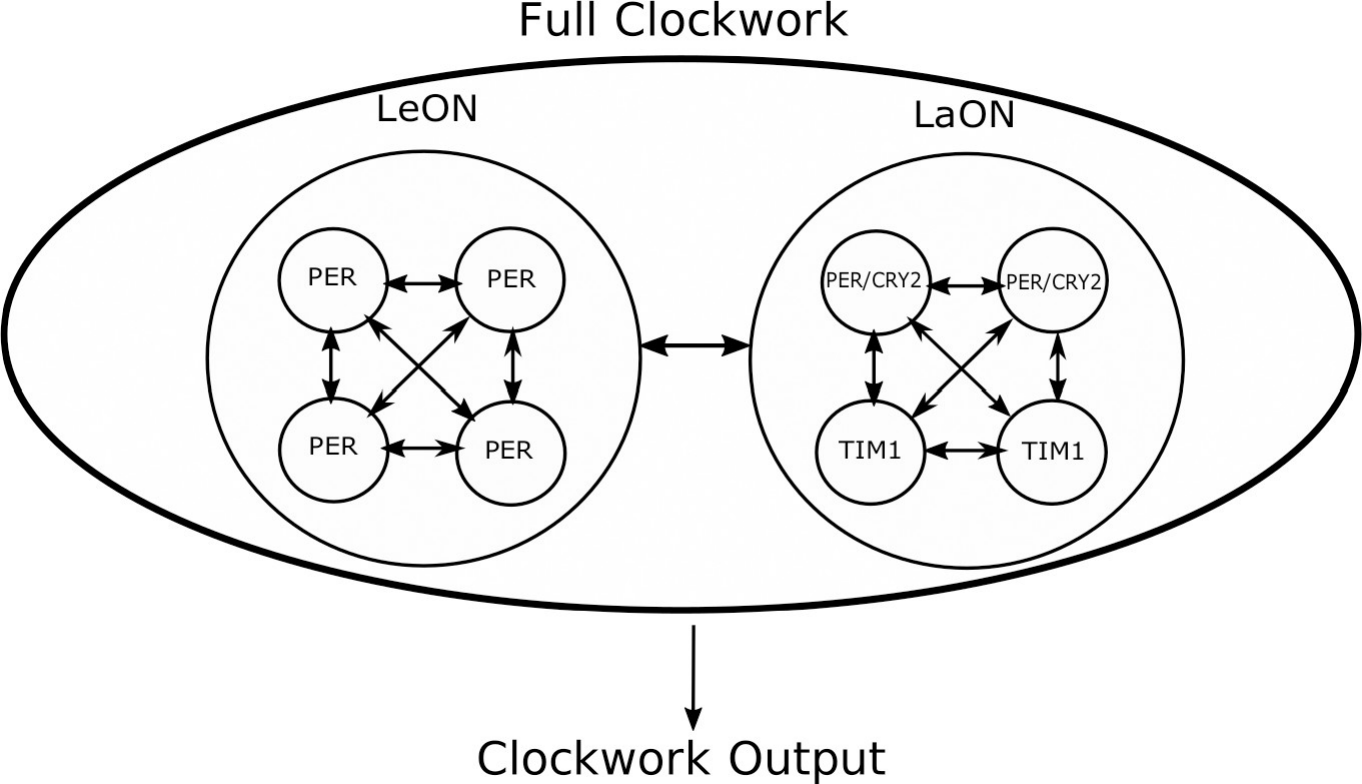
Two circadian oscillator networks with different molecular clockworks per accessory medulla control locomotor activity rhythms of the Madeira cockroach. The lead oscillator network (LeON) with a shorter period and the lag oscillator network (LaON) with a longer period, each consist of four single oscillator neurons. Both networks synchronize with each other and, together, they control locomotor activity rhythms.

We wanted to know whether all results obtained in the RNA_i_ experiments described before could be explained with the assumption that two different circadian oscillator networks per AME comprising of clock cells with either short (lead oscillator network = LeON; τ<24h) or long τ (lag oscillator network = LaON, τ>24h) control locomotor activity rhythms. Since knockdown of CRY2 and of TIM1 cause period shortening of locomotor activity rhythms both molecules must be part of LaON. Since PER knockdown does not change the period, PER is assumed to be part of both LeON and LaON. Thus, while LeON comprises cells that express PER as transcriptional inhibitor, LaON consists of two cell types that either express PER/CRY2,or TIM1 alone as transcriptional repressors. Since our experiments demonstrated that CRY2 and PER depend on each other, we assumed that CRY2 can enter the nucleus only together with PER. However, since CRY2 knockdown elevated mRNA levels of PER, PER can enter the nucleus alone and more efficiently blocks transcription than together with CRY2. Also, TIM1 can enter the nucleus without the need to heteromerize with other clock proteins. Accordingly, a mathematical model was developed that describes the dynamics of the cockroach clockwork as observed in the knockdown experiments (Figs 10–12). The model is based on the conjecture that two separate oscillator networks exist in the clockwork, interacting with each other to determine the duration of the cockroach´s daily locomotor activity rhythms in response to external triggers such as dusk or dawn stimuli. In the clockwork structure suggested here LeON and LaON coexist, both expressing individual periods. Depending on the interaction between the two networks and on the exposition to external triggers, the complete clockwork expresses shorter or longer periods. In the nominal case, i.e. when no knockdown is imposed and the insect is exposed to constant darkness, a balance between the LeON and the LaON determines a period of around 24 h.

To start modeling the full clockwork, a single oscillator is first introduced as a building block. Such a single oscillator represents the dynamics of the oscillating concentration of a quantity being relevant to the circadian rhythm within a single cell. As opposed to the majority of previous work relying on single oscillator models of the Goodwin type [30], such an oscillator is here modeled as a Switching Linear System (SLS), which is a subclass of so called Hybrid Automata (Fig 10), which combine continuous and discrete-valued dynamics. See e.g. Henzinger et al. [31] for a definition of hybrid automata and Bortolussi et al. [32] for an overview of the use of more hybrid systems in system biology. The reason of starting from SLS for the clockwork model in this paper is their advantage over Goodwin models with respect to much easier parameterization to obtain desired oscillating behavior, and easier analysis of periods, phases, and synchronization of coupled models. Already in 1978 Glass and Pasternack [33] compared Goodwin models to piecewise linear models. Goodwin models are helpful if the details of transcriptional inhibitions are known since they include directly highly nonlinear inhibition terms. Piecewise linear models are useful to parameterize easily measured features such as oscillation periods. Since biochemical details of transcriptional inhibition are not available in the cockroach we apply in our manuscript the analytically treatable SLS approach. To establish the single cell oscillator, an SLS model is defined as follows:

Definition 1—Switching Linear System: *Given a partition of a real-valued state space* $X\subseteq R^{n}$ *into polyhedra X*_*i*_ = {*x*∈X|C_i_*x*≤*d*_*i*_), *i*∈{1,2,…,*n*_*z*_}, *and an assignment of linear dynamics* $\dot{x}(t)=A_{i}∙x(t)+B_{i}∙u(t)$ *with matrices* $A_{i}\in R^{n\times n}$ *and* $B_{i}\in R^{m\times m}$ *to any X*_*i*_, *where* $u(t)\in R^{m}$ *denotes system inputs*. *The evolution of the state x*(*t*)∈*X over time* t∈R *starting in x*(0)∈*X then follows for u*(*t*) = 0 *from a sequence of phases* [*t*_*k*_, *t*_*k*+1_] *bounded by switching times* $t_{k}\in R,\;k\in \{0,1,2,\ldots \},\;t_{0}=0$ *with solutions* $x(t)=e^{A_{i}(t-t_{k})}∙x(t_{k})$, *where A_i_ is selected by x*(*t*)∈*X*_*i*_ *for t*∈[*t*_*k*_, *t*_*k*+1_].

While the inputs allow us to model light stimuli *u*(*t*) = 0 is selected in the above definition, since the SLS in this paper is used to model experiments where the insects are kept in constant darkness. Oscillating behavior of an SLS can be obtained by appropriate choice of the pairs (*X*_*i*_,*A*_*i*_). For example, the simple SLS with *n*_*z*_ = 2, *n* = 3, and *X*_1_ = {*x*|0≤*x*_1_, 0≤*x*_2_≤10} and *X*_2_ = {*x*|0≤*x*_1_, 10≤*x*_2_} as well as $A_{1}=\left[\begin{array}{ccc} 1 & 0 & 0\\ 1 & -1 & 0\\ 1 & 0 & -1 \end{array}\right]$ and $A_{2}=\left[\begin{array}{ccc} -1 & 0 & 0\\ 2 & -1 & 0\\ 1 & 0 & -1 \end{array}\right]$ determines a single oscillator **(**Fig 10) for *x*(0) = [10,10,10]^*T*^. A possible biological interpretation is that the states model the concentrations of mRNA (*x*_1_), protein (*x*_2_), and a coupling substance (*x*_3_), while *x*_2_ acts as an inhibitor of *x*_1_.

To couple several single oscillators to a network, more precisely to the LeON and the LaON respectively, the following approach is taken: The LeON is established exemplarily by n_Le_ = 4 single oscillators, representing four cells in which only PER appears (Fig 11). Note that the number of oscillators in LeON may vary, but is chosen small here for illustration purposes.

The SLS of the cells with index *j* ∈ {1,2,3,4} modeling the LeON are as follows, where $\tau_{Le}^{\left(j\right)}$ is a factor to scale the state-dependent part of each oscillator (thus to allow for heterogeneity):

$$\begin{array}{c} {\dot{x}}_{Le}^{\left(j\right)}(t)=\tau_{Le}^{\left(j\right)}A_{i}x_{Le}^{\left(j\right)}(t)+E_{Le,i}\cdot K_{Le}(t),x_{Le}^{\left(j\right)}(t)\in X_{i}^{\left(j\right)}\\ K_{Le}(t)=\frac{1}{n_{Le}}\sum_{l=1}^{n_{Le}}x_{Le,3}^{\left(l\right)}(t). \end{array}$$

The second term models the coupling of the cell oscillations, represented by the mean field *K*_*Le*_(t) over the third states of the single oscillators in the *n*_*Le*_ = 4 cells, similarly as in Gonze et al. (2005) [34], and $E_{Le,i}\in R^{n\times 1}$ specifies the coupling strength. The complete LeON is parameterized to determine a period of the circadian rhythm of less than 24 h.

The LaON is also chosen to comprise n_La_ = 4 single oscillators, two of them representing cells in which TIM1 is contained, while the remaining two refer to cells with PER/CRY2(right part of Fig 11). With index *h* ∈ {1,2,3,4} and the same coupling structure as for LeON, the model of the LaON is given by:

$$\begin{array}{c} {\dot{x}}_{La}^{\left(h\right)}(t)=\tau_{La}^{\left(h\right)}A_{i}∙x_{La}^{\left(h\right)}(t)+E_{La,i}\cdot K_{La}(t),x_{La}^{\left(h\right)}(t)\in X_{i}^{\left(h\right)}\\ K_{La}(t)=\frac{1}{n_{La}}\sum_{l=1}^{n_{La}}x_{La,3}^{\left(l\right)}(t) \end{array}$$

By merging all oscillators contained in LaON (or those, respectively, in LeON) into a single model, again a model of type SLS is obtained. To obtain the dynamic representation of the full clockwork, the interaction of LeON and LaON needs to be represented, where bidirectional coupling is proposed here (Fig 12).

Also for the coupling of LeON and LaON, a mean field structure with coupling factors *F*_*Le*,*i*_, $F_{La,i}\in R^{n\times n}$ is chosen, leading to a full model according to:

$${\dot{x}}_{Le}^{\left(j\right)}(t)=\tau_{Le}^{\left(j\right)}A_{i}∙x_{Le}^{\left(j\right)}(t)+E_{Le,i}\cdot K_{Le}(t)+F_{Le,i}\cdot K_{La}(t),x_{Le}^{\left(j\right)}(t)\in X_{i}^{\left(j\right)}$$

$${\dot{x}}_{La}^{\left(h\right)}(t)=\tau_{La}^{\left(h\right)}A_{i}∙x_{La}^{\left(h\right)}(t)+E_{La,i}\cdot K_{La}(t)+F_{La,i}\cdot K_{Le}(t),x_{La}^{\left(h\right)}(t)\in X_{i}^{\left(h\right)}$$

$$K_{Le}(t)=\frac{1}{n_{Le}}\sum_{j=1}^{n_{Le}}x_{Le,3}^{\left(j\right)}(t),K_{La}(t)=\frac{1}{n_{La}}\sum_{j=1}^{n_{La}}x_{La,3}^{\left(h\right)}(t),\;K(t)=0.5∙(K_{Le}(t)+K_{La}(t))$$

**Fig 13:**
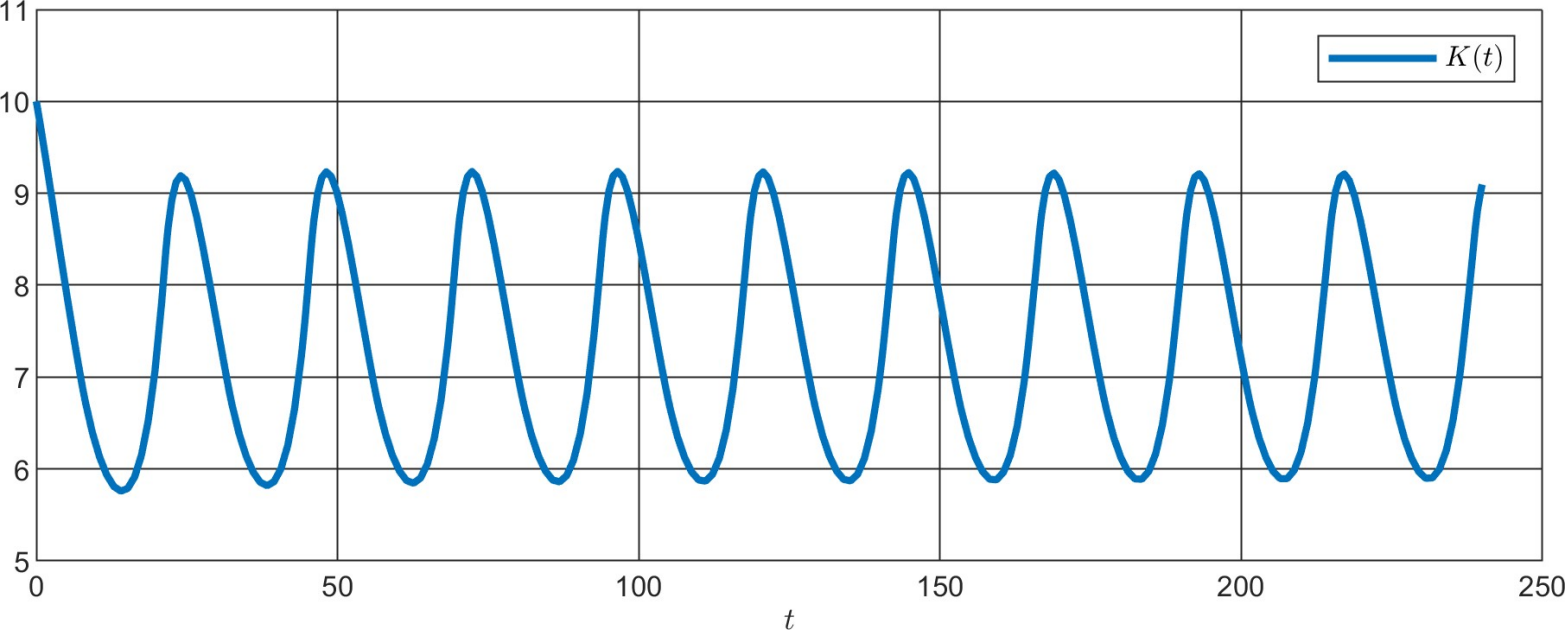
Simulation of the output K(t) of the nominal clockwork with a period of 23.60 h.

The variable *K*(t) represents the overall output of the full clockwork. The matrices *A*_*i*_ and the switching surface of each single oscillator are selected as in the single oscillator example above. The other model parameters are chosen to: *E*_*Le*,1_ = −*E*_*Le*,2_ = [0.07 0 0]^*T*^, *E*_*La*,1_ = *E*_*La*,2_ = [0.07 0 0]^*T*^, *F*_*Le*,1_ = −*F*_*Le*,2_ = [−0.05 0 0]^*T*^, *F*_*La*,1_ = *F*_*La*,2_ = [−0.12 0 0]^*T*^. Fig 13 shows the course of *K*(t) over time for a simulation of the nominal clockwork, revealing that the networks (LeON and LaON) synchronize with a common period of 23.6 h.

### Modeling and simulation of the gene knockdown experiments

Based on the model described above, gene knockdown experiments can simply be simulated by eliminating those oscillators, which are affected by the knockdown at all respective times. The reduction of the number of oscillators for three knockdown simulations are listed (Table 3).

**Table 3:** Reduction of the number of oscillators as respective knockdown simulations.

Knockdown of:	*n*_*Le*_	*n*_*La*_
**None**	4	4
**PER**	1	2
**TIM1**	4	2
**CRY2**	4	2

**Fig 14:**
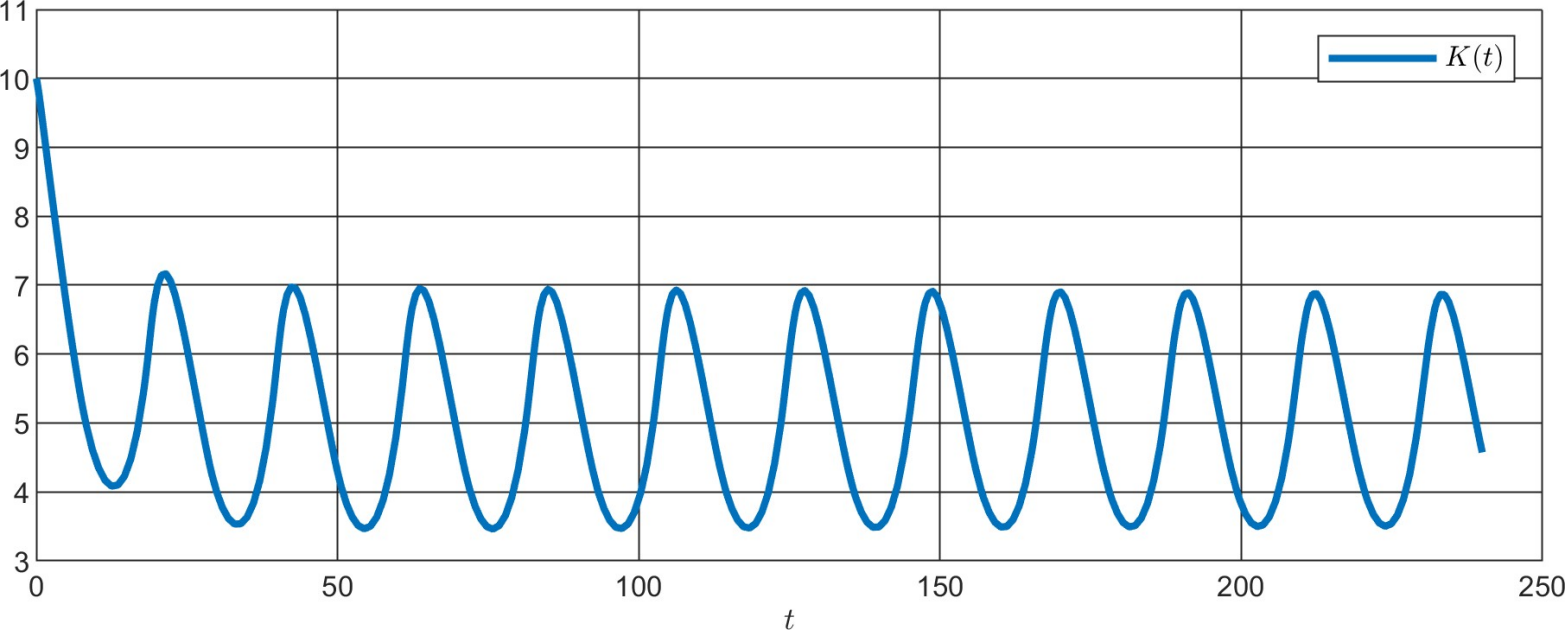
Simulation of K(t) for TIM1 or CRY2 knockdown with a period of 20.50 h.

**Fig 15:**
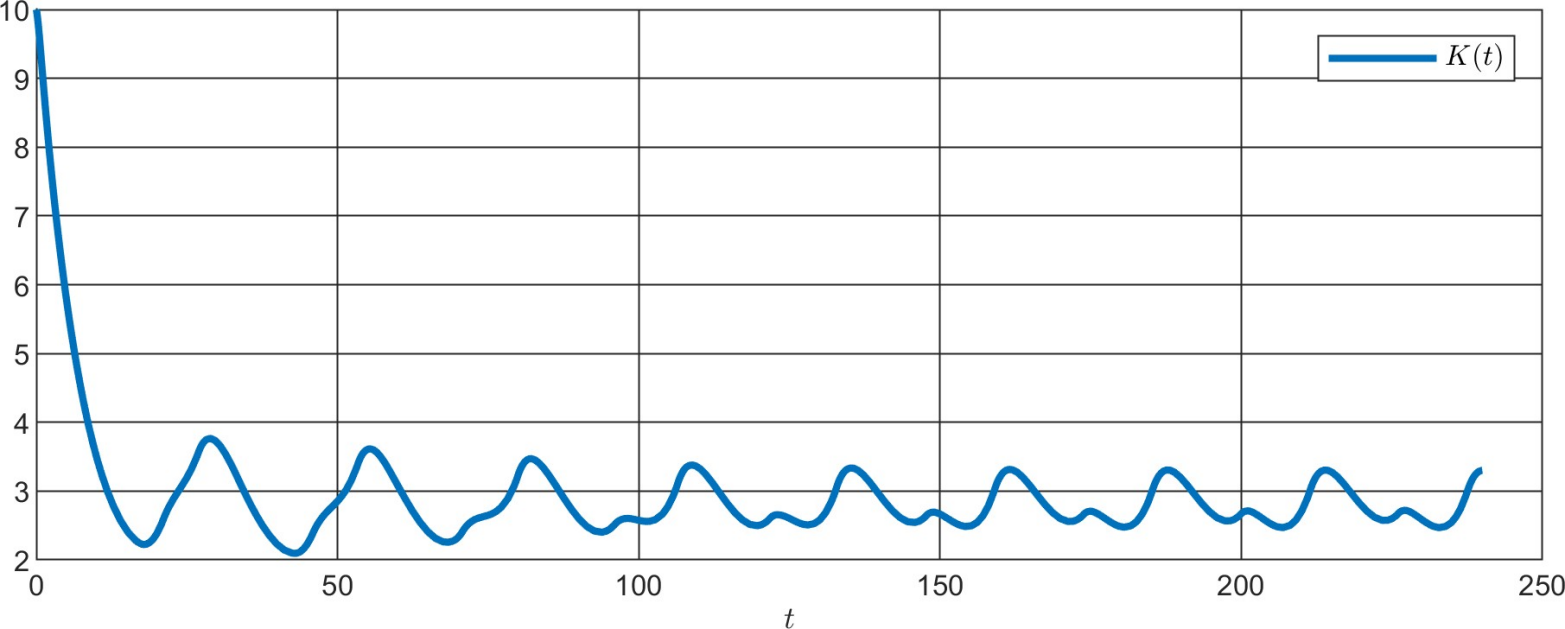
Simulation of K(t) for PER knockdown with a period of 25.96 h.

Since for TIM1 and CRY2 knockdown the number of oscillators eliminated are the same (Table 3), simulation results for both cases are qualitatively the same as shown (Fig 14). The plot demonstrates that after a transient phase the output *K(t)* and, thus, the oscillator networks regain synchrony, while the period is reduced to 20.5 h, in contrast to simulation of PER knockdown. Here, almost the complete LeON is eliminated, consisting of one oscillator, while in the LaON two oscillators are left (Fig 15). The complete clockwork still synchronizes, but in contrast to our experimental data the period becomes longer in comparison to the nominal case. Thus, our model predicts that LeON comprises of an additional repressor of core clock genes next to PER.

## Discussion

With RNA interference (RNA_i_)-dependent knockdown of mRNA levels of the circadian clock genes *Rm´per*, *Rm´tim1*,and *Rm´cry2* of the Madeira cockroach we examined which of these negative feedback loops of the core feedback loop of the circadian clockwork are indispensable for circadian locomotor activity rhythms. While this method does not allow for a complete knock-out of a gene product, nevertheless, it is well established that decreasing concentrations of gene products compromised their functions. Unexpectedly, we found that neither depletion of Rm´PER or Rm´TIM1, nor depletion of Rm´CRY2 alone deleted circadian locomotor activity rhythms in constant conditions, independent of the strength of the knockdown. Furthermore, while knockdown of *Rm´per* mRNA did not significantly change the period (τ) of circadian locomotor activity rhythms in rhythmically remaining cockroaches, for both other RNA_i_ experiments the circadian periods of circadian locomotor rhythms were shortened significantly. Based upon these unexpected results we developed a hypothesis to explain our data on a cellular level comprising clock neurons with different period and different core feedback loops. To formulate a very basic quantitative model of the cockroach molecular clockwork that allows to challenge this hypothesis, we employed a system of ordinary differential equations. In contrast to most other published oscillator models, here an oscillator was modelled as a Switching Linear System (SLS), for which parametrization is relatively easy to obtain the oscillatory behavior as observed in the experiments. The data were modeled by groups of cells establishing two coupled feedback loops with CLK/CYC as the positive- and either PER alone, PER/CRY2, or TIM1 alone as the negative feedback of the loop. We hypothesized that two different coupled ensembles of circadian clock neurons control circadian locomotor activity rhythms that comprise at least three different clock cell types with different core clockworks in the Madeira cockroach. One cell type contains PER, another contains PER/CRY2 heterodimers as negative regulators of transcription, while the third employs TIM1 but neither PER nor CRY2. We assumed that a leading oscillator ensemble (the *morning oscillator*) expressing PER controls a short period rhythm in constant darkness, while PER/CRY2 or only TIM1 expressing neurons belong to a lagging oscillator network (the *evening oscillator*) which controls a long period locomotor activity rhythm. Challenging our hypothesis with modelling predicted that next to PER there must be at least one additional negative regulator in the leading oscillator network in the Madeira cockroach.

### Different insect species differ in their core circadian clockworks

New molecular techniques such as transcriptomics and RNA_i_ allowed for the molecular analysis of non-model insect species. While knockout of genes during development usually triggers compensatory mechanisms to maintain homeostasis, the acute knockdown of specific mRNAs allows for acute downregulation of the protein in question. Since RNA_i_ can be differentially successful revealing different extends of concentration decreases it is possible to reveal the importance of protein concentration for particular physiological mechanisms, as powerful molecular mechanism of physiological analysis.

Unexpectedly, experiments revealed that there are quite some differences in the molecular feedback loops of circadian clockworks between different insect species. While the positive elements of the core feedback loop, CLK and CYC were present in all insect species examined, either one or the other, or both were rhythmically expressed [4]. While in *D*. *melanogaster Dm´clk* is rhythmically expressed, *Dm´cyc* is constitutively expressed [2]. In mammals such as mice it is vice versa: the CYC homolog BMAL1 is rhythmically expressed, while CLK is present at constant levels [35]. Interestingly, in the cricket *G*.*bimaculatus* it was found that while Gb´CLK is required, Gb´CYC is dispensable for rhythmic locomotor activity rhythms and rhythmic expression of the clock genes *Gb´per* and *Gb´tim* [36, 14]. So far, amongst the non-model insects the molecular clockwork of crickets was studied best [4]. Surprisingly, the relevance of specific clock genes differed between the hemimetabolous cricket *G*. *bimaculatus* and the hemimetabolous Madeira cockroach *R*. *maderae*. While for the cricket PER is necessary for rhythmic locomotor activity rhythms [37], it is dispensable for the cockroach. Furthermore, the roles of TIM1 and CRYs for the expression of circadian locomotor activity rhythms differ between both species. While in both crickets and cockroaches CRY2 is expressed, considerably more is known about the respective functions in crickets as compared to cockroaches [8, 29, 38]. While in our current experiments knockdown of *Rm´cry2* only shortened locomotor activity rhythms, for the crickets longer and shorter periods could occur next to loss of rhythmicity [29]. Furthermore, while in *R*. *maderae* Rm´CRY2 appeared to interact with Rm´PER but not with Rm´TIM1, in *G*. *bimaculatus* different splice forms of Gb´CRY2 appeared to interact with each other or with Gb´CRY1, but not with Gb´TIM. In contrast, Gb´TIM interacted with Gb´PER, forming an independently cycling negative feedback loop. Therefore, there are differences and redundancy in the general scheme of the negative feedback loops between insect species, even when the same clock molecules are being expressed. Since in a circadian clock neuron that controls rhythmic behavior a circadian molecular clockwork is a prerequisite to obtain circadian rhythmicity, we assume that in the Madeira cockroach there are different clock neurons expressing different molecular feedback loops.

### The accessory medulla (AME) with pigment-dispersing factor (PDF) neurons is the circadian clock that controls circadian rest-activity cycles in the cockroach

Lesion and transplantation experiments located the circadian clock of the Madeira cockroach that controls rest activity cycles to the AME with PDF processing neurons as its outputs to locomotor control areas [39, 40]. The AME is innervated by seven adjacent soma groups that are abundant of colocalized neuropeptides [41, 42]. The evolutionary conserved PDF is the best studied among these circadian neuropeptides. In *Drosophila* PDF is important for synchronized rhythms of circadian clock gene expression, for regular circadian sleep-wake rhythms in constant darkness, and for light-controlled adjustment to long photoperiods [43, 44]. Also in the Madeira cockroach it appears to serve the same functions as in *Drosophila* [45, 13]. Cockroach PDF clock neurons are located in an anterior (aPDFMEs) and a posterior soma group (pPDFMEs) next to the AME. Among them are four contralateral aPDFMEs that project to the contralateral optic lobe directly connecting both bilaterally symmetric AMEs as circadian coupling pathway. In addition, contralaterally projecting clock neurons such as contralateral aPDFMEs are clock outputs connecting the clock to locomotor control centers [12, 46–48, 39, 49–52]. Thus, it was suggested that contralaterally projecting aPDFMEs control locomotor activity rhythms, while ipsilaterally remaining aPDFMEs control sleep/rest in the Madeira cockroach [53, 54].

### The circadian pacemaker system of the Madeira cockroach consists of PDF-dependent dual oscillator circuits controlling sleep-wake cycles

The crepuscular fruitfly *D*. *melanogaster* is active at dusk and dawn, expressing a bimodal activity pattern [55, 44]. In contrast, the nocturnal Madeira cockroach rests during the day and is active during the night [47]. However, dependent on the light conditions, the unimodal nocturnal activity pattern of the cockroach can dissociate into a bimodal pattern peaking at dusk and dawn, reminiscent of *Drosophila* [56]. In the fruit fly, two neuronal circuits termed morning (M) and evening (E) oscillators control the two peaks of the crepuscular locomotor activity rhythms [44, 55, 57–59]. The PDF-releasing and PDF-sensitive small ventrolateral neurons (sLNvs) are M cells that control a short period of the locomotor rhythm which is locked onto dawn. In contrast, E cells express PDF-receptors, but not the neuropeptide PDF and control a long period locomotor rhythm locked onto dusk [60–65]. There are at least three groups of E cells (E1-3) that process distinct neuropeptides and serve different, not yet well discerned functions [66–68].However, as postulated in the cockroach also in *Drosophila* sleep controlling neuronal circuits differ from locomotor activity controlling circuits [13, 55, 65, 69]. While M cells in *Drosophila* express and also sense PDF, its E cells only sense PDF. In the cockroach, we hypothesized that different PDF-sensing and -expressing AME neurons take part in both M and E circuits [53]. Based upon a strong correlation between branching patterns and PDF sensitivity [53] we proposed that ipsilateral branching PDF-sensitive M cells promote rest, while contralateral PDF-sensitive E cells were suggested to promote activity. Based upon our current experiments, we hypothesize that ipsilateral PDFMEs are LeON mediating short τ locomotor rhythms. They are proposed to express a molecular clockwork with PER as negative transcription regulators. In contrast, two ensembles of E cells such as contralateral aPDFMEs are LaON. They are suggested to express either TIM1 alone, or PER/CRY2 in the transcriptional/posttranscriptional feedback loop. Modeling of our hypothesis was able to simulate our data only partially. Since PER knockdown in the model obtained synchrony only together with strong period lengthening, but experiments only revealed a tendency to lengthen periods, it is possible that there is an additional negative transcription regulator next to Rm´PER. Possibly, also the respective not complete RNA_i_-dependent decrease in mRNA levels was responsible for a lack of significant period lengthening. Future experiments will challenge this new hypothesis of different core feedback loops in different M and E oscillator cells in the Madeira cockroach and will examine whether there is an additional *Rm´per* gene in the Madeira cockroach.

## Supporting information

S1 Fig Injection site of the dsRNA. Green food dye was added in this injection to demonstrate fluid distribution. In the actual experiments, no food dye was used.
(PNG)

S1 Table Primers. (XLSX)

S2 Table Analysis of rhythmicity in behavioral experiments. (XLSX)

S3 Table Analysis of period in behavioral experiments. (XLSX)

S4 Table Analysis of mRNA levels in behavioral experiments. (XLSX)

S5 Table Analysis of mRNA levels in time series. (XLSX)

## References

[1] Michel S, Meijer JH. From clock to functional pacemaker. Eur J Neurosci 2019. 10.1111/ejn.14388 PMC7027845 30793396

[2] Hardin PE. Chapter 5—Molecular Genetic Analysis of Circadian Timekeeping in *Drosophila* In: Brody S, editor. Advances in Genetics: The Genetics of Circadian Rhythms. Academic Press; 2011 p. 141–73 Available from: URL: http://www.sciencedirect.com/science/article/pii/B9780123876904000052. 10.1016/B978-0-12-387690-4.00005-2 PMC4108082 21924977

[3] Michael AK, Fribourgh JL, van Gelder RN, Partch CL. Animal Cryptochromes: Divergent Roles in Light Perception, Circadian Timekeeping and Beyond. Photochem Photobiol 2017; 93(1):128–40. 10.1111/php.12677 27891621 PMC5397253

[4] Tomioka K, Matsumoto A. Circadian molecular clockworks in non-model insects. Current Opinion in Insect Science 2015; 7:58–64. 10.1016/j.cois.2014.12.006 32846680

[5] Rubin EB, Shemesh Y, Cohen M, Elgavish S, Robertson HM, Bloch G. Molecular and phylogenetic analyses reveal mammalian-like clockwork in the honey bee (*Apis mellifera*) and shed new light on the molecular evolution of the circadian clock. Genome Res 2006; 16(11):1352–65. 10.1101/gr.5094806 17065608 PMC1626637

[6] Benna C, Bonaccorsi S, Wülbeck C, Helfrich-Förster C, Gatti M, Kyriacou CP et al *Drosophila timeless2* Is Required for Chromosome Stability and Circadian Photoreception. Current Biology 2010; 20(4):346–52. Available from: URL: http://www.sciencedirect.com/science/article/pii/S0960982209022118. 10.1016/j.cub.2009.12.048 20153199

[7] Danbara Y, Sakamoto T, Uryu O, Tomioka K. RNA interference of timeless gene does not disrupt circadian locomotor rhythms in the cricket *Gryllus bimaculatus*. Journal of Insect Physiology 2010; 56(12):1738–45. Available from: URL: http://www.sciencedirect.com/science/article/pii/S0022191010002131. 10.1016/j.jinsphys.2010.07.002 20637213

[8] Werckenthin A, Derst C, Stengl M. Sequence and expression of *per*, *tim1*, and *cry2* genes in the Madeira cockroach *Rhyparobiamaderae*. J Biol Rhythms 2012; 27(6):453–66. Available from: URL: https://journals.sagepub.com/doi/full/10.1177/0748730412462109. 23223371 10.1177/0748730412462109

[9] Tomioka K, Matsumoto A. A comparative view of insect circadian clock systems. Cell Mol Life Sci 2010; 67(9):1397–406. 10.1007/s00018-009-0232-y 20035363 PMC11115600

[10] Sehgal A, Price JL, Man B, Young MW. Loss of circadian behavioral rhythms and *per* RNA oscillations in the *Drosophila* mutant *timeless*. Science 1994; 263(5153):1603–6. 10.1126/science.8128246 8128246

[11] Kamae Y, Tomioka K. *timeless* is an essential component of the circadian clock in a primitive insect, the firebrat *Thermobia domestica*. J Biol Rhythms 2012; 27(2):126–34. 10.1177/0748730411435997 22476773

[12] Page TL. Transplantation of the cockroach circadian pacemaker. Science 1982; 216(4541):73–5. 10.1126/science.216.4541.73 17809802

[13] Stengl M, Arendt A. Peptidergic circadian clock circuits in the Madeira cockroach. Curr Opin Neurobiol 2016; 41:44–52. 10.1016/j.conb.2016.07.010 27575405

[14] Uryu O, Kamae Y, Tomioka K, Yoshii T. Long-term effect of systemic RNA interference on circadian clock genes in hemimetabolous insects. Journal of Insect Physiology 2013; 59(4):494–9. Available from: URL: http://www.sciencedirect.com/science/article/pii/S0022191013000474. 10.1016/j.jinsphys.2013.02.009 23458340

[15] Livak KJ, Schmittgen TD. Analysis of relative gene expression data using real-time quantitative PCR and the 2^-ΔΔCT^ Method. Methods 2001; 25(4):402–8. 10.1006/meth.2001.1262 11846609

[16] van der Walt S, Colbert SC, Varoquaux G. The NumPy Array: A Structure for Efficient Numerical Computation. Comput. Sci. Eng. 2011; 13(2):22–30.

[17] Virtanen P, Gommers R, Oliphant TE, Haberland M, Reddy T, Cournapeau D et al SciPy 1.0—Fundamental Algorithms for Scientific Computing in Python; 2019 7 23 Available from: URL: http://arxiv.org/pdf/1907.10121v1. 10.1038/s41592-019-0686-2 PMC7056644 32015543

[18] Hunter JD. Matplotlib: A 2D Graphics Environment. Comput. Sci. Eng. 2007; 9(3):90–5.

[19] Wickham H, Averick M, Bryan J, Chang W, McGowan L, François R et al Welcome to the Tidyverse. JOSS 2019; 4(43):1686.

[20] reticulate. Version 1.14; 2019. Available from: URL: https://github.com/rstudio/reticulate.

[21] xsp. Version 0.1.2; 2017. Available from: URL: https://CRAN.R-project.org/package=xsp.

[22] nlme: Linear and Nonlinear Mixed Effects Models. Version 3.1–145; 2020. Available from: URL: https://CRAN.R-project.org/package=nlme.

[23] Hothorn T, Bretz F, Westfall P. Simultaneous Inference in General Parametric Models. Biometrical Journal 2008; (50(3)):346–63. 10.1002/bimj.200810425 18481363

[24] Lenth RV. Least-Squares Means: The R Package lsmeans. J. Stat. Soft. 2016; 69(1).

[25] Boisgontier MP, Cheval B. The anova to mixed model transition. Neurosci Biobehav Rev 2016; 68:1004–5. 10.1016/j.neubiorev.2016.05.034 27241200

[26] Lindstrom MJ, Bates DM. Newton—Raphson and EM Algorithms for Linear Mixed-Effects Models for Repeated-Measures Data. Journal of the American Statistical Association 1988; 83(404):1014–22.

[27] Laird NM, Ware JH. Random-Effects Models for Longitudinal Data. Biometrics 1982; 38(4):963 7168798

[28] Tomioka K, Sakamoto T, Moriyama Y. RNA interference is a powerful tool for chronobiological study in the cricket. Sleep and Biological Rhythms 2009; 7(3):144–51. Available from: URL: 10.1111/j.1479-8425.2009.00407.x.

[29] Tokuoka A, Itoh TQ, Hori S, Uryu O, Danbara Y, Nose M et al *cryptochrome* genes form an oscillatory loop independent of the *per/tim* loop in the circadian clockwork of the cricket *Gryllus bimaculatus*. Zoological Lett 2017; 3(1):1–14. Available from: URL: https://zoologicalletters.biomedcentral.com/track/pdf/10.1186/s40851-017-0066-7. 28405468 10.1186/s40851-017-0066-7 PMC5383941

[30] Komin N, Murza AC, Hernández-García E, Toral R. Synchronization and entrainment of coupled circadian oscillators. Interface Focus 2011; 1(1):167–76. 10.1098/rsfs.2010.0327 22419982 PMC3262239

[31] Henzinger TA. The Theory of Hybrid Automata In: Davies SP, editor. Verification of digital and hybrid systems. [Place of publication not identified]: Springer; 2012 p. 265–92 Available from: URL: 10.1007/978-3-642-59615-5_13.

[32] Bortolussi L, Policriti A. Hybrid Systems and Biology In: Formal Methods for Computational Systems Biology: 8^th^ International School on Formal Methods for the Design of Computer, Communication, and Software Systems, SFM 2008 Bertinoro, Italy, June 2–7, 2008 Advanced Lectures. Berlin, Heidelberg: Springer-Verlag Berlin Heidelberg; 2008 p. 424–48 [Lecture Notes in Computer Science; vol. 5016].

[33] Glass L, Pasternack JS. Stable oscillations in mathematical models of biological control systems. J. Math. Biology 1978; 6(3):207–23.

[34] Gonze D, Bernard S, Waltermann C, Kramer A, Herzel H. Spontaneous Synchronization of Coupled Circadian Oscillators. Biophysical Journal 2005; 89(1):120–9. Available from: URL: http://www.sciencedirect.com/science/article/pii/S0006349505726643. 10.1529/biophysj.104.058388 15849258 PMC1366510

[35] Takahashi JS. Transcriptional architecture of the mammalian circadian clock. Nat Rev Genet 2017; 18(3):164–79. Available from: URL: https://www.nature.com/articles/nrg.2016.150.pdf. 10.1038/nrg.2016.150 27990019 PMC5501165

[36] Moriyama Y, Kamae Y, Uryu O, Tomioka K. *Gb’Clock* Is Expressed in the Optic Lobe and Is Required for the Circadian Clock in the Cricket *Gryllus bimaculatus*. J Biol Rhythms 2012; 27(6):467–77. 10.1177/0748730412462207 23223372

[37] Moriyama Y, Sakamoto T, Karpova SG, Matsumoto A, Noji S, Tomioka K. RNA interference of the clock gene *period* disrupts circadian rhythms in the cricket *Gryllus bimaculatus*. J Biol Rhythms 2008; 23(4):308–18. 10.1177/0748730408320486 18663238

[38] Kutaragi Y, Tokuoka A, Tomiyama Y, Nose M, Watanabe T, Bando T et al A novel photic entrainment mechanism for the circadian clock in an insect: involvement of *c-fos* and *cryptochromes*. Zoological Lett 2018; 4(1):1–12. Available from: URL: https://zoologicalletters.biomedcentral.com/track/pdf/10.1186/s40851-018-0109-8. 30250749 10.1186/s40851-018-0109-8 PMC6145112

[39] Stengl M, Homberg U. Pigment-dispersing hormone-immunoreactive neurons in the cockroach *Leucophaea maderae* share properties with circadian pacemaker neurons. Journal of Comparative Physiology A 1994; 175(2):203–13. Available from: URL: 10.1007/BF00215116. 8071895

[40] Reischig T, Stengl M. Ectopic transplantation of the accessory medulla restores circadian locomotor rhythms in arrhythmic cockroaches (*Leucophaea maderae*). Journal of Experimental Biology 2003a; 206(11):1877–86. 12728009 10.1242/jeb.00373

[41] Petri B, Stengl M, Würden S, Homberg U. Immunocytochemical characterization of the accessory medulla in the cockroach *Leucophaea maderae*. Cell Tissue Res 1995; 282(1):3–19. 10.1007/BF00319128 8581923

[42] Reischig T, Stengl M. Ultrastructure of pigment-dispersing hormone-immunoreactive neurons in a three-dimensional model of the accessory medulla of the cockroach *Leucophaea maderae*. Cell Tissue Res 2003b; 314(3):421–35. Available from: URL: 10.1007/s00441-003-0772-7. 14557869

[43] Helfrich-Förster C. The period clock gene is expressed in central nervous system neurons which also produce a neuropeptide that reveals the projections of circadian pacemaker cells within the brain of *Drosophila melanogaster*. Proc Natl Acad Sci U S A 1995; 92(2):612–6. 10.1073/pnas.92.2.612 7831339 PMC42792

[44] Helfrich-Förster C. From neurogenetic studies in the fly brain to a concept in circadian biology. J Neurogenet 2014; 28(3–4):329–47. 10.3109/01677063.2014.905556 24655073

[45] Stengl M, Werckenthin A, Wei H. How does the circadian clock tick in the Madeira cockroach? Current Opinion in Insect Science 2015; 12:38–45.

[46] Page TL. Regeneration of the optic tracts and circadian pacemaker activity in the cockroach *Leucophaea maderae*. Z. Vergl. Physiol. 1983a; 152(2):231–40.

[47] Page TL. Circadian organization in cockroaches: Effects of temperature cycles on locomotor activity. Journal of Insect Physiology 1985; 31(3):235–42. Available from: URL: http://www.sciencedirect.com/science/article/pii/0022191085901258.

[48] Page TL, Caldarola PC, Pittendrigh CS. Mutual entrainment of bilaterally distributed circadian pacemaker. Proc Natl Acad Sci U S A 1977; 74(3):1277–81. 10.1073/pnas.74.3.1277 265571 PMC430667

[49] Reischig T, Stengl M. Morphology and pigment-dispersing hormone immunocytochemistry of the accessory medulla, the presumptive circadian pacemaker of the cockroach *Leucophaea maderae*: a light- and electron-microscopic study. Cell Tissue Res 1996; 285(2):305–19.

[50] Reischig T, Stengl M. Optic lobe commissures in a three-dimensional brain model of the cockroach *Leucophaea maderae*: a search for the circadian coupling pathways. J Comp Neurol 2002; 443(4):388–400. 10.1002/cne.10133 11807846

[51] Reischig T, Petri B, Stengl M. Pigment-dispersing hormone (PDH)-immunoreactive neurons form a direct coupling pathway between the bilaterally symmetric circadian pacemakers of the cockroach *Leucophaea maderae*. Cell Tissue Res 2004; 318(3):553–64. 10.1007/s00441-004-0927-1 15578273

[52] Söhler S, Stengl M, Reischig T. Circadian pacemaker coupling by multi-peptidergic neurons in the cockroach *Leucophaea maderae*. Cell Tissue Res 2011; 343(3):559–77. 10.1007/s00441-010-1091-4 21229364 PMC3046342

[53] Gestrich J, Giese M, Shen W, Zhang Y, Voss A, Popov C et al Sensitivity to Pigment-Dispersing Factor (PDF) Is Cell-Type Specific among PDF-Expressing Circadian Clock Neurons in the Madeira Cockroach. J Biol Rhythms 2018; 33(1):35–51. 10.1177/0748730417739471 29179611

[54] Arnold T, Korek S, Massah A, Eschstruth D, Stengl M. Candidates for photic entrainment pathways to the circadian clock via optic lobe neuropils in the Madeira cockroach. J Comp Neurol 2020. 10.1002/cne.24844 31860126

[55] Helfrich-Förster C. Does the morning and evening oscillator model fit better for flies or mice? J Biol Rhythms 2009; 24(4):259–70. 10.1177/0748730409339614 19625728

[56] Schendzielorz T. Analysis of second messengers in peripheral and central circadian pacemakers [Dissertation]. Kassel: Universität Kassel; 2014.

[57] Kistenpfennig C, Nakayama M, Nihara R, Tomioka K, Helfrich-Förster C, Yoshii T. A Tug-of-War between Cryptochrome and the Visual System Allows the Adaptation of Evening Activity to Long Photoperiods in *Drosophila melanogaster*. J Biol Rhythms 2018; 33(1):24–34. 10.1177/0748730417738612 29179610

[58] Yoshii T, Rieger D, Helfrich-Förster C. Chapter 4—Two clocks in the brain: An update of the morning and evening oscillator model in *Drosophila* In: Kalsbeek A, Merrow M, Roenneberg T, Foster RG, editors. Progress in Brain Research: The Neurobiology of Circadian Timing. Elsevier; 2012 p. 59–82 Available from: URL: http://www.sciencedirect.com/science/article/pii/B9780444594273000277. 10.1016/B978-0-444-59427-3.00027-7 22877659

[59] Yoshii T, Hermann-Luibl C, Helfrich-Förster C. Circadian light-input pathways in *Drosophila*. Commun Integr Biol 2016; 9(1):e1102805 10.1080/19420889.2015.1102805 27066180 PMC4802797

[60] Grima B, Chélot E, Xia R, Rouyer F. Morning and evening peaks of activity rely on different clock neurons of the *Drosophila* brain. Nature 2004; 431(7010):869–73. 10.1038/nature02935 15483616

[61] Stoleru D, Peng Y, Agosto J, Rosbash M. Coupled oscillators control morning and evening locomotor behaviour of *Drosophila*. Nature 2004; 431(7010):862–8. 10.1038/nature02926 15483615

[62] Yoshii T, Funada Y, Ibuki-Ishibashi T, Matsumoto A, Tanimura T, Tomioka K. *Drosophila cry*^*b*^ mutation reveals two circadian clocks that drive locomotor rhythm and have different responsiveness to light. Journal of Insect Physiology 2004; 50(6):479–88. Available from: URL: http://www.sciencedirect.com/science/article/pii/S0022191004000356. 10.1016/j.jinsphys.2004.02.011 15183277

[63] Rieger D, Shafer OT, Tomioka K, Helfrich-Förster C. Functional analysis of circadian pacemaker neurons in *Drosophila melanogaster*. J Neurosci 2006; 26(9):2531–43. 10.1523/JNEUROSCI.1234-05.2006 16510731 PMC6793667

[64] Dolezelova E, Nothacker H-P, Civelli O, Bryant PJ, Zurovec M. A *Drosophila* adenosine receptor activates cAMP and calcium signaling. Insect Biochem Mol Biol 2007; 37(4):318–29. Available from: URL: http://www.sciencedirect.com/science/article/pii/S0965174806002542. 10.1016/j.ibmb.2006.12.003 17368195

[65] Helfrich-Förster C. Sleep in Insects. Annu Rev Entomol 2018; 63:69–86. 10.1146/annurev-ento-020117-043201 28938081

[66] Liang X, Ho MCW, Zhang Y, Li Y, Wu MN, Holy TE et al Morning and Evening Circadian Pacemakers Independently Drive Premotor Centers via a Specific Dopamine Relay. Neuron 2019; 102(4):843–857.e4. 10.1016/j.neuron.2019.03.028 30981533 PMC6533154

[67] Yao Z, Shafer OT. The *Drosophila* circadian clock is a variably coupled network of multiple peptidergic units. Science 2014; 343(6178):1516–20. 10.1126/science.1251285 24675961 PMC4259399

[68] Schubert FK, Hagedorn N, Yoshii T, Helfrich-Förster C, Rieger D. Neuroanatomical details of the lateral neurons of *Drosophila melanogaster* support their functional role in the circadian system. J Comp Neurol 2018; 526(7):1209–31. 10.1002/cne.24406 29424420 PMC5873451

[69] Goda T, Tang X, Umezaki Y, Chu ML, Kunst M, Nitabach MN et al *Drosophila* DH31 Neuropeptide and PDF Receptor Regulate Night-Onset Temperature Preference. J Neurosci 2016; 36(46):11739–54. 10.1523/JNEUROSCI.0964-16.2016 27852781 PMC5125228

