# Supplementary figures and images for: Neither per, nor tim1, nor cry2 alone are essential components of the molecular circadian clockwork in the Madeira cockroach
Source: PLoS One. 2020 Aug 4;15(8):e0235930. doi: 10.1371/journal.pone.0235930 (PMC7402517; doi:10.1371/journal.pone.0235930)

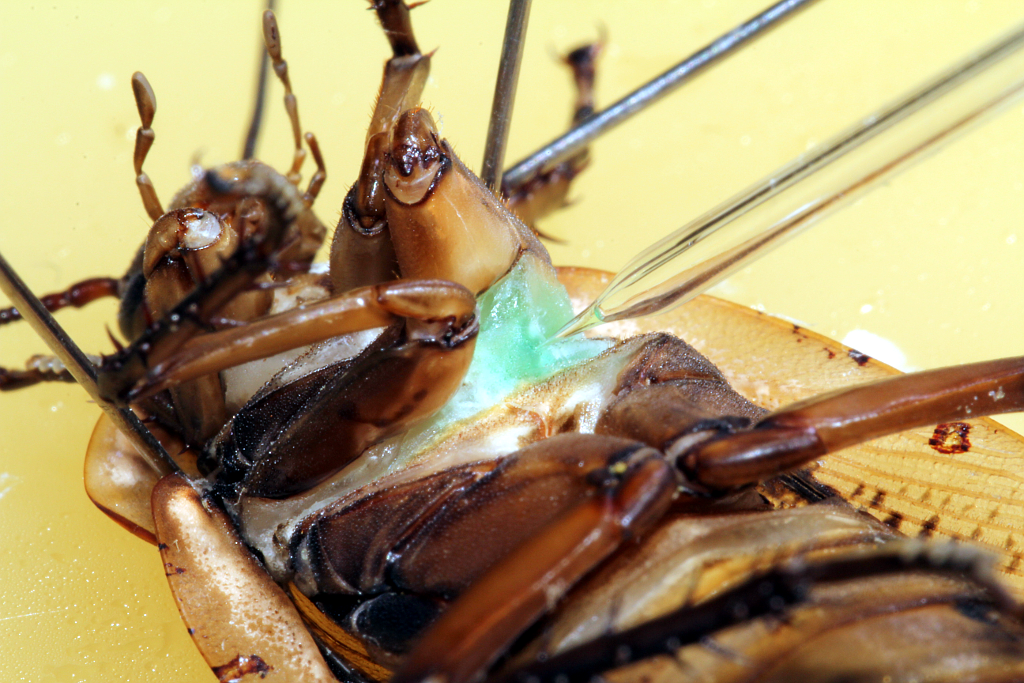

Supplement: S1 Fig — Green food dye was added in this injection to demonstrate fluid distribution. In the actual experiments, no food dye was used. (PNG) [file pone.0235930.s001.png]
